# *In Vitro* Mutational and Bioinformatics Analysis of the M71 Odorant Receptor and Its Superfamily

**DOI:** 10.1371/journal.pone.0141712

**Published:** 2015-10-29

**Authors:** Jaclyn Bubnell, Sophie Jamet, Delia Tomoiaga, Charlotte D’Hulst, Konstantinos Krampis, Paul Feinstein

**Affiliations:** 1 Department of Biological Sciences, Hunter College, CUNY, New York, NY, United States of America; 2 The Graduate Center Biology Program, CUNY, New York, NY, United States of America; 3 The Graduate Center Behavioral and Cognitive Neuroscience Program, CUNY, New York, NY, United States of America; 4 Director of Bioinformatics, Center for Translational and Basic Research, CUNY, New York, NY, United States of America; Monell Chemical Senses Center, UNITED STATES

## Abstract

We performed an extensive mutational analysis of the canonical mouse odorant receptor (OR) M71 to determine the properties of ORs that inhibit plasma membrane trafficking in heterologous expression systems. We employed the use of the M71::GFP fusion protein to directly assess plasma membrane localization and functionality of M71 in heterologous cells *in vitro* or in olfactory sensory neurons (OSNs) *in vivo*. OSN expression of M71::GFP show only small differences in activity compared to untagged M71. However, M71::GFP could not traffic to the plasma membrane even in the presence of proposed accessory proteins RTP1S or mβ2AR. To ask if ORs contain an internal “kill sequence”, we mutated ~15 of the most highly conserved OR specific amino acids not found amongst the trafficking non-OR GPCR superfamily; none of these mutants rescued trafficking. Addition of various amino terminal signal sequences or different glycosylation motifs all failed to produce trafficking. The addition of the amino and carboxy terminal domains of mβ2AR or the mutation Y289A in the highly conserved GPCR motif NPxx**Y** does not rescue plasma membrane trafficking. The failure of targeted mutagenesis on rescuing plasma membrane localization in heterologous cells suggests that OR trafficking deficits may not be attributable to conserved collinear motifs, but rather the overall amino acid composition of the OR family. Thus, we performed an *in silico* analysis comparing the OR and other amine receptor superfamilies. We find that ORs contain fewer charged residues and more hydrophobic residues distributed throughout the protein and a conserved overall amino acid composition. From our analysis, we surmise that it may be difficult to traffic ORs at high levels to the cell surface *in vitro*, without making significant amino acid modifications. Finally, we observed specific increases in methionine and histidine residues as well as a marked decrease in tryptophan residues, suggesting that these changes provide ORs with special characteristics needed for them to function in olfactory neurons.

## Introduction

Since the first odorant receptor (OR) was cloned in 1991, major goals in the field of olfaction have been to characterize and identify ligands for odorant receptors, to understand how olfactory function affects human health, and to identify the mechanisms underlying olfactory perception. These goals are still very far from being achieved. Although ORs make up the largest family of seven transmembrane receptors (7TM) in the human genome, almost all of the human odorant receptors are functionally uncharacterized [1].

Odorant receptors are located on the cilia of olfactory sensory neurons (OSNs) and bind odorants from the environment, which leads to G-protein-mediated activation of the cyclic AMP pathway. Excluding allelic variants, there are over 1000 intact OR coding sequences in mice, and 350 in humans; each type of OR protein theoretically responds to a subset of odorants delivered at low concentrations [2–4]. An effective high-throughput system of analysis is required to understand the molecular basis of odor activation of this vast family of receptors. Progress in the field of olfaction has been hindered by the lack of effective *in vitro* expression systems for ORs. Compared to *in vivo* systems, *in vitro* systems provide a rapid and cost-effective means of analyzing the structure and the function of proteins.

Expressing ORs in heterologous cells has been met with minimal success due to endoplasmic reticulum (ER) retention of OR proteins. There has been some progress in trafficking small amounts of receptor protein to the cell surface *in vitro*, but the receptor is often forced out to the plasma membrane by signal leader sequence fusions, ignoring the properties that are obstructing trafficking [5, 6]. These forcibly expressed ORs are not likely to be properly folded and may not provide accurate ligand-binding profiles.

Moreover, for the past 20 years, many laboratories have used complementation assays to identify proteins expressed in OSNs that would allow ORs to traffic out of the ER in heterologous cells. “Accessory proteins”, such as Receptor-Transporting Protein 1 and 2 (RTP1; RTP2) and Receptor Expression Enhancing Protein (REEP), help to traffic some ORs to the plasma membrane *in vitro* when they are co-expressed with an OR [7]. Both of these protein types are natively and specifically expressed in olfactory neurons and interact with ORs. Although there is evidence that ligand-binding profiles for ORs co-expressed with RTP and REEP are similar to those *in vivo*, these accessory proteins do not enable plasma membrane expression for all ORs [7]. Even more, the level of plasma membrane expression for each OR when co-expressed with RTPs differs greatly and the successfully addressed ORs are usually tagged at the amino terminus. This is a step forward in the search for factors involved in OR trafficking, but it is far from a solution. However, insight into the mechanisms preventing ORs from plasma membrane expression could be provided by closely examining the interactions between accessory proteins and ORs.

Virtually all members of the OR superfamily tested either poorly traffic or show no trafficking to the plasma membrane *in vitro* if they are expressed without cofactors. In contrast, other G-Protein Coupled Receptors (GPCRs) like the canonical β_2_-Adrenergic Receptor (β2AR) robustly traffic to the plasma membrane in heterologous cells [8]. We have begun to break down the biological barriers that are preventing plasma membrane expression of ORs by understanding how the β2AR traffics to the plasma membrane [9].

Here, we have developed an assay to analyze OR trafficking by generating OR Carboxy-terminal (Ct) fluorescent protein fusions and expressing them in a cell line derived from the mouse olfactory placode, OP6. Previous studies have shown that the fusion of the green fluorescent protein (GFP) does not affect GPCR trafficking and ligand activation ([10]; [9]). We have previously observed that the Ct fusion of GFP to the M71 OR (M71::GFP) neither affects its trafficking to the cilia nor to the axons when expressed *in vivo* [11]. Here, we further validate the functionality of M71::GFP by demonstrating its odorant sensitivity is nearly identical to M71 when expressed in OSNs. In contrast, M71::GFP does not traffic to the plasma membrane in heterologous cells.

We attempted to “fix M71” trafficking through extensive targeted mutagenesis and chimeric analyses with the mouse β2AR (mβ2AR); we generated 29 mutants and co-expressed them with RTP1S *in vitro*. The mutations target a range of OR sequences including conserved regions between ORs not present in β2AR, N-linked glycosylation site modifications, and the addition of signal leader sequences. Finally, we generated chimeras with β2AR and mutated a conserved region (NPxxY; Y289A mutation) involved in mβ2AR trafficking [9]. None of the mutations rescues plasma membrane trafficking.

These results indicate that the failure of ORs to traffic to the plasma membrane *in vitro* might be a distributed effect of the amino acid composition. We performed a bioinformatic analysis that reveals the OR superfamily has fewer charged residues and more hydrophobic residues than non-OSN expressed amine or adrenergic receptors. Thus, there may not be a single region of the OR protein, but rather a broad distribution of residues, which prevents functional expression of ORs to the plasma membrane in heterologous cells.

## Material and Methods

### Plasmid construction

All constructs used in this study are listed in S1 Text. We used an *in vitro* expression vector for the GPCR fusion constructs (D346) and the gap protein fusion constructs, as previously described [10]. We codon optimized the M71 coding sequence for mouse to improve protein expression in heterologous cells and used this sequence for all mutagenesis and chimeric analyses. The mβ2AR coding sequence is from mouse strain 129 cDNA, as previously described [12]. RTP1S was amplified from mouse olfactory cDNA with 5’ aaaagagctcaagcttcgaattcggcgcgccaccatgtgtaagagtgtga 3’ and 5’ ttaattaatcagacagaagtacggaaggagaat 3’ primers, shuttled into the pGemT Easy vector (Promega).

We generated mutations and chimeric constructs using the following methods: for point mutations we used primers designed for QuickChange mutagenesis (Stratagene), and all other constructs and mutants were generated through PCR amplification and pGemT Easy ligation, or through Gibson Assembly cloning directly into the D346 expression vector (New England Biolabs Inc). Constructs in the pGemT vector were subcloned into the *in vitro* expression vector described above as EcoRI/PacI cassettes to generate GFP or mCherry GPCR fusions. See S1 Text for nucleotide and amino acid sequences and their plasmid names.

### OP6 cell culture and transfection

Mouse olfactory placode cells were maintained (OP6 [13], a gift from Jane Roskams) as previously described [8] in Dulbecco’s modified Eagle’s medium (DMEM 1X Gibco) supplemented with 10% fetal bovine serum (FBS Gibco) and 1% penicillin/streptomycin (Pen Strep Millipore) at 33°C. We transiently transfected plasmid DNA constructs using the Amaxa Nucleofector (Lonza) with PBS at 60%-70% confluency according to the manufacturer’s protocol. Transfected cells were allowed to recover for 24 hours at 33°C and express the plasmid DNA before imaging.

### Laser scanning confocal microscopy

We live-imaged OP6 cells on a Zeiss LSM 510 microscope using a Zeiss Plan-APO 25X water immersion objective in culture media. We excited GFP at 488 nm and collected at BP500-545 nm mCherry at 561 nm and collected at LP575 nm. All images were acquired using the multi-tracking feature.

### Filopodia counts

We counted fluorescently labeled filopodia as a measure of plasma membrane trafficking as described in [9]. To stain the plasma membrane, we used Cellmask Deep Red Plasma Membrane reagent (Life Technologies) as described in [9].

### Dose response to 2,4 dimethyl acetophenone

We performed the dose response assay by Calcium-based Fluorometric Imaging Plate Reader (FLIPR) as previously described [8].

### Electrophysiological recordings

Patch clamp recordings on OSN knobs was performed on epithelial explants as previously described [14]. The Institutional Animal Care and Use Committee at Northwestern University specifically approved these studies.

### Bioinformatics

Logo plots were generated using the programs Clustal Omega (http://www.ebi.ac.uk/Tools/msa/clustalo/) for multiple sequence alignment and WebLogo 3 (http://weblogo.threeplusone.com/create.cgi). GPCR protein sequences were obtained from the GPCR database (http://www.gpcr.org/7tm/). Our analysis of GPCRs was restricted to those with lengths between 270–401 residues starting from the most amino terminal N-linked glycosylation motif, NxS/T/C to the NPxxY at the end of transmembrane 7. See raw data (S1 Table) and accepted/discarded sequences (S1 File).

## Results

### A GFP fusion to the C-terminus of M71 minimally affects its functionality *in vivo*

There are few GPCRs that have been well characterized as N-terminal (Nt) or C-terminal (Ct) GFP fusion proteins. We previously expressed *in vitro*, mβ2AR::GFP or various fluorescent protein (XFP) Ct fusion proteins in heterologous cell lines. All versions of the mβ2AR::XFP Ct had the same EC50 to a single high affinity agonist, isoprenaline as compared to the untagged mβ2AR. No other pharmacological characterization of the mβ2AR::GFP fusion protein has been performed either *in vitro* nor *in vivo* (no gene-targeted nor transgenic mice exist). In contrast, we have previously characterized the M71 OR protein (olfr151) fused to GFP *in vivo*. M71::GFP is capable of localizing to olfactory cilia and axons as well as promoting axon outgrowth, axon identity, and glomerular formation in the mouse olfactory system [11]. However, we did not compare its *in vivo* odorant response profile to the untagged M71 protein.

In 2003, only two M71 agonists had been identified, acetophenone and benzaldehyde. Acetophenone proved to be a more reliable agonist with an EC50 of ~19.6μM. In 2012, we described additional odorants that could specifically activate M71, but not its 96% homologous paralogue M72 (olfr160). These odorants include ligands with higher maximal activations and EC50s: 2-amino acetophenone (2aACP), 4-methyl acetophenone (4mACP), piperonal (PIP), and ethyl maltol (eMLT) [14].

**Fig 1:**
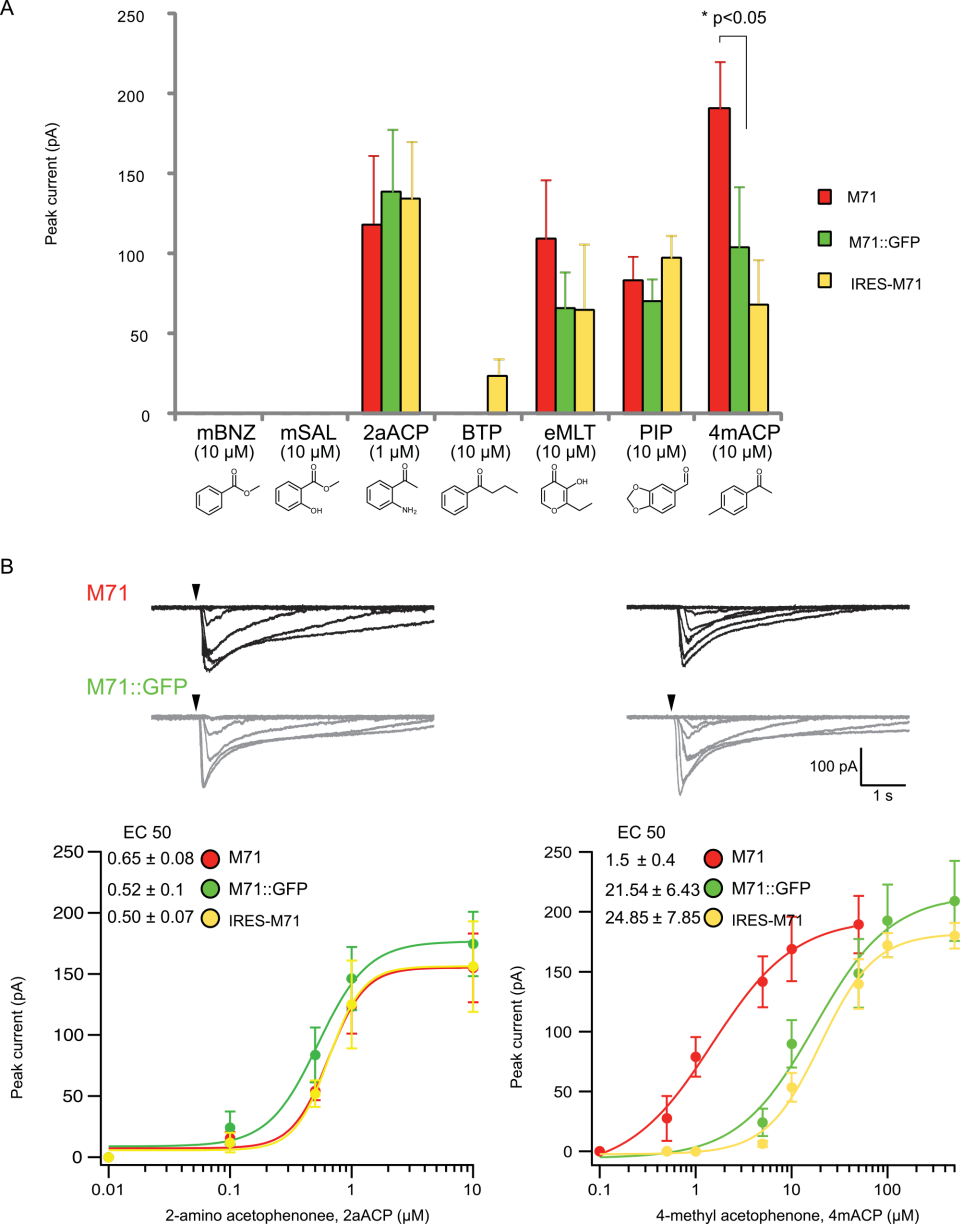
*Ex vivo* profiling of M71::GFP fusion protein. (A) Peak currents for M71 (red), M71::GFP (green) and IRES-M71 (yellow) extracellular dendritic knobs after excitation by seven odorants: five M71 specific odorants with variable affinities (mBNZ:benzaldehyde, 2aACP:2-amino acetophenone, eMLT:ethyl maltol, PIP:piperinal, 4mACP:4methyl acetophenone) and two M72 specific odorants (mSal: methyl salicylate and BTP:butryophenone). Odorants arranged by structure. Concentration for six odorants were all at 10μM except 2aACP was 1μM due to its higher affinity. The major difference observed was 2 fold higher peak current to 4mACP for M71 relative to both M71::GFP and IRES-M71. (B) Current traces for M71 and M71::GFP show similar desensitization profiles to 2aACP and 4mACP regardless of 4mACP 1 log lower EC50. Single cell dose response curves for M71 (red), M71::GFP (green) and IRES-M71 (yellow) reveal a 1 log difference lower in EC50 to 4mACP relative to M71.

We reveal that olfactory neurons expressing either M71 or M71::GFP are activated by M71 specific ligands and not by M72 specific ligands methyl salicylate(mSal) and butyrophenone (BTP) (Fig 1A). In addition, the low affinity M71 ligand, benzaldehyde (EC50 ~98μM), activated neither M71 nor M71::GFP at 10μM. In contrast, the highest affinity M71 ligand, 2-amino acetophenone had nearly identical EC50s (0.65± 0.08 vs. 0.52± 0.1 μM) for M71 and M71::GFP (Fig 1B). However, for 4-methyl acetophenone the M71::GFP EC50 was 10x lower than M71 (1.5± 0.4 vs. 21.54± 6.43 μM). These results suggest that M71::GFP ligand binding specificity has been mostly unaltered. However, there was a difference in 4-methyl acetophenone responsiveness, which might be explained by lower expression levels of the M71::GFP in the plasma membrane compared to M71. Support for this proposal comes from our previously reported M71 mutant that has an apparent 3-fold reduction in M71 levels in olfactory cilia, also causes a 10-fold decrease in 4-methylacetophenone responsiveness without affecting the 2-amino acetophenone EC50 (Fig 1A and 1B). These data show that the M71::GFP fusion protein retains an EC50 and response profile for the highest-affinity M71 odors that appear refractory to receptor protein levels and the GFP Ct moiety.

### GPCR::GFP plasma membrane trafficking using the GFP-filopodia assay

To study heterologous expression of ORs we employed a cell line derived from the mouse olfactory placode (OP). OP6 cells are OSN precursors and possess numerous filopodia most likely due to their neuronal origins. We have previously shown that when a cytoplasmic fluorescent protein is expressed in OP6 cells, few or no filopodia are fluorescently labeled. On the contrary, when the fluorescent protein is fused to proteins that successfully traffic to the plasma membrane, the protein locates to filopodia [10]. Thus, in order to quickly and accurately assess the plasma membrane localization of GPCRs, we assay for the presence of GPCRs fused to the green fluorescent protein (GPCR::GFP) within the filopodia of OP6 cells. Localization of this fusion protein to the filopodia indicates successful trafficking to the plasma membrane and the lack of labeling at the filopodia indicates non-satisfactory trafficking to the plasma membrane [9].

**Fig 2:**
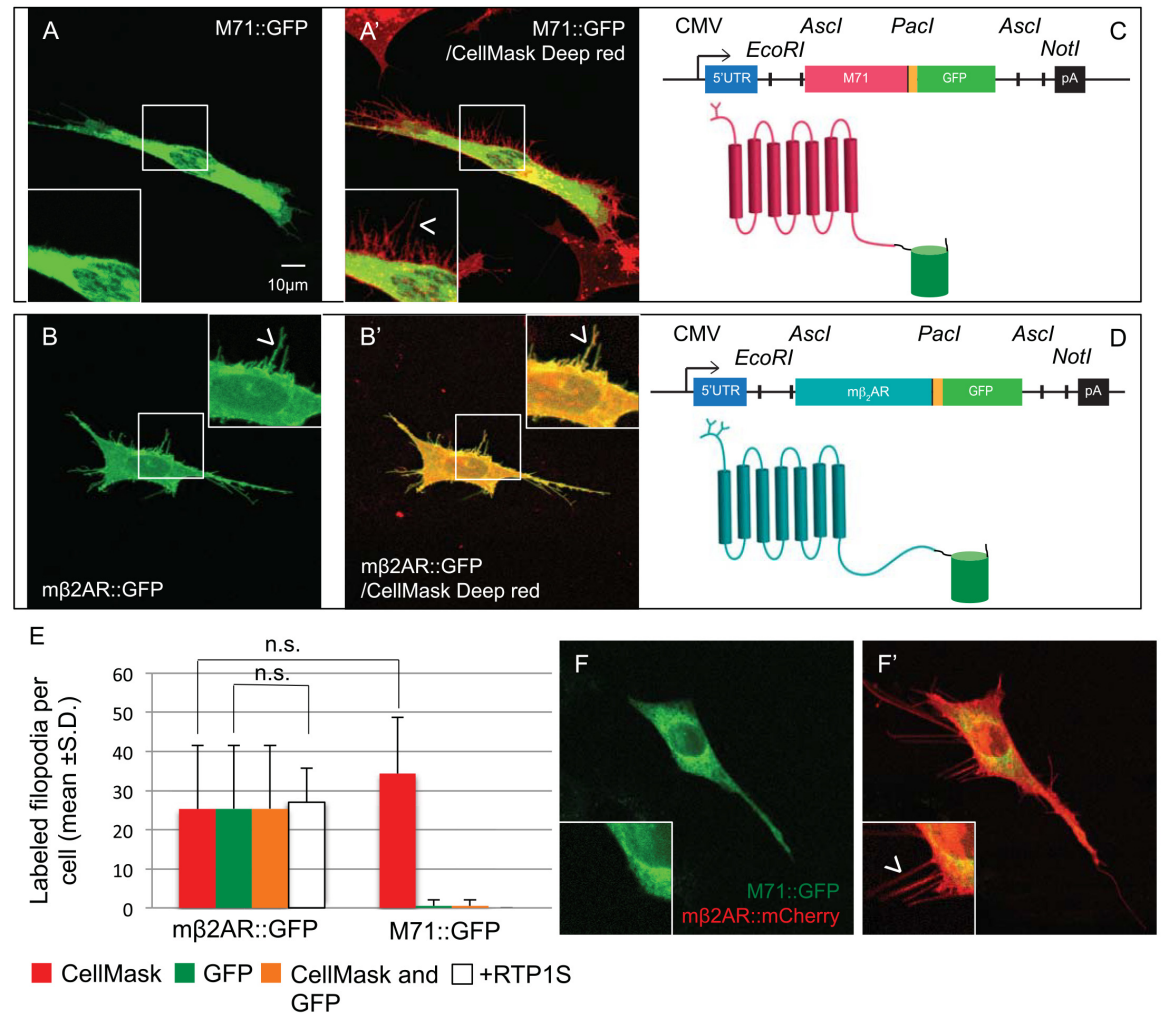
M71::GFP does not traffic to the plasma membrane. (A) M71::GFP expression in OP6 cells in trapped perinuclearly (A’) Same cell with colabelling of plasma membrane revealed by CellMask Red. (B) mβ2AR::GFP expression in OP6 cells gives a flat morphology that has sharp focus. Expression is observed in filopodia. (B’) Same cell as (B) with colabelling of plasma membrane revealed by CellMask Red. (C, D) M71::GFP and mβ2AR::GFP expression vectors. (E) Filopodia counts by Cell Mask Red and GFP for mβ2AR::GFP and mβ2AR::GFP. (F) M71::GFP co-expression with mβ2AR::mCherry in OP6 cells maintains GFP fluorescence trapped perinuclearly whereas (F’) mβ2AR::mCherry expression is found in the filopodia.

We chose this OR as a preliminary example to dissect OR trafficking *in vitro*, because of the wealth of *in vivo* ligands for M71. When we express M71::GFP in OP6 cells, no filopodia are labeled with GFP and the receptor is localized perinuclearly (Fig 2A, 2C and 2E). On the contrary, when we express mβ2AR::GFP in OP6 cells, it localizes to the filopodia with an average of 32 filopodia per cell across 10 cells, and thereby reflecting successful trafficking to the plasma membrane (Fig 2B, 2D and 2E). To confirm the presence of filopodia in our transfected cells, we stained the plasma membrane of OP6 cells with CellMask Deep Red Plasma membrane stain, and observed the same number of filopodia for cells expressing either M71::GFP (33.8 ±13.7 filopodia per cell) or mβ2AR::GFP (25.5 ±16.0 filopodia per cell). This result suggests that the expression of different GPCR::GFP does not affect the number of filopodia in OP6 cells (Fig 2E). As expected, mβ2AR::GFP expression in the filopodia is almost always co-labeled with CellMask but there is almost no detectable GFP fluorescence in the filopodia of M71::GFP expressing cells: 25.4 ±16.1 double-labeled filopodia for mβ2AR::GFP vs. 0.6 ±1.6 for M71::GFP (Fig 2B’ and 2D’).

Co-expression of M71::GFP with the short form of the OR accessory protein, RTP1 (RTP1S), reveals no labeled filopodia, confirming accounts from other labs that RTP1S does not always rescue plasma membrane trafficking of the M71 OR [7]. Not surprisingly, when mβ2AR::GFP is co-expressed with RTP1S, there was no difference in the number of labeled filopodia, 27 vs. 32 per cell for 10 cells (Fig 2E).

Previous studies have suggested that co-expression of M71 and β2AR improves M71 trafficking [15]. When we co-express M71::GFP and mβ2AR::mCherry, the M71 OR localizes perinuclearly, whereas the mβ2AR successfully traffics to the plasma membrane and labels filopodia (Fig 2F and 2F’). Thus, in the same cell in which mβ2AR::mCherry successfully traffics to the plasma membrane and M71::GFP does not, providing a good internal control that individual OP6 cells retain the capacity to properly traffic GPCRs. These results indicate that retention of M71 in the ER does not prevent β2AR trafficking to the plasma membrane and mβ2AR is not a cofactor for the trafficking of M71.

### Conserved OR superfamily specific residues do not prevent M71 plasma membrane trafficking

**Fig 3:**
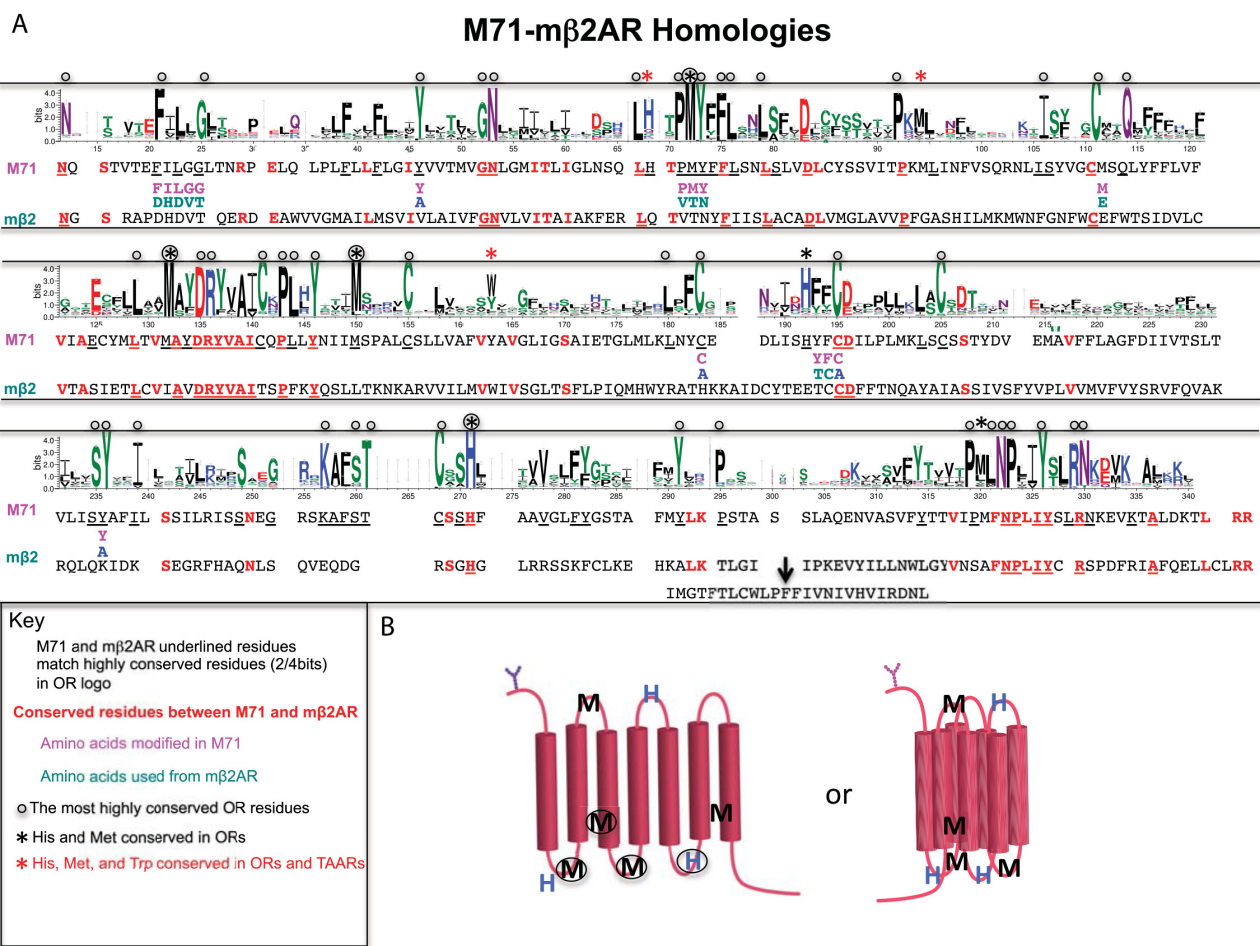
Conserved OR sequences revealed by logo plot shared with M71 and mβ2AR. (A) OR logo using mouse Class I and II odorant receptor sequences. Most highly conserved OR residues are depicted with black circles. M71 and mβ2AR sequences are delineated underneath logo plot; Residues with greater than 50% bit conservation to OR logo are underlined. Conserved residues between M71 and mβ2AR are in bold and red. M71 residue swaps between M71 and mβ2AR are delineated in purple and green, respectively. M71 residues converted to alanine are described with bold blue A. Five conserved methionine residues, three conserved histidine residues and one weakly conserved tryptophan residue marked with asterisk. Two of these residues are also found in OSN expressed TAARs marked with red asterisk. (B) Schematic of OR seven transmembrane structure with conserved methionine and histidine residues depicted in linear and predicted 3 dimensional state. Methionine and histidine residues in non-transmembrane might be in close enough proximity to coordinate a metal group, like copper. Methionine residues in TM3 and TM7 may form bridge if modified.

To better understand what prevents OR expression in heterologous cells, we performed a multiple sequence alignment (MSA) using Clustal Omega of OR predicted proteins to look at conserved residues that might play a role in the retention of ORs in the ER. An OR logo plot for the MSA depicts sparse conservation (Fig 3A; Near 100% conservation is distributed to a handful of residues in OR Logo plot; 47/302 residues; see also [16]). The overall protein homology between the M71 OR and the mβ2AR is very poor, even though the mβ2AR can act as a surrogate OR *in vivo* (18%: 56/302 residues; 5% is considered random, which would be 15/302 residues). The M71 and the mβ2AR conservation within the transmembrane domains is sparse, 16/56 residues (Fig 3A; TM1, GN residues; TM3, DRYVAI residues; and in TM3, NPLIY residues)[11]. These residues also are conserved within the OR superfamily.

We wanted to determine if there were sequences specific to ORs that caused their ER retention. Logo plots between GPCRs, ORs and vomeronasal receptors (V1Rs) were compared to identify residues that are conserved across non-chemosensory GPCRs but have different conservations in either ORs or V1Rs. We mutated sequences conserved only amongst ORs or V1Rs and not found in mβ2AR. In addition, we mutated selected regions of M71 that differed from mβ2AR; Residues in M71 sequences were either replaced with those of the mβ2AR or alanine.

M71::GFP mutants were subsequently tested in our GFP-labeled filopodia assay for plasma membrane expression in OP6 cells. The first group of mutations were as follows: FILGG (residues 12–16) to DHDVT, Y35A, PMY(residues 58–60) to VTN, M98E, C169A, YF (residues 176–177) to TC, C178A, and Y217A. These mutations targeted the OR superfamily conserved residues: FxLxG12-16, PMY58-60, Y35, Y217A and residues involved in the formation of disulfide bridges between conserved cysteines: M98, C169, and YF176-177.

**Fig 4:**
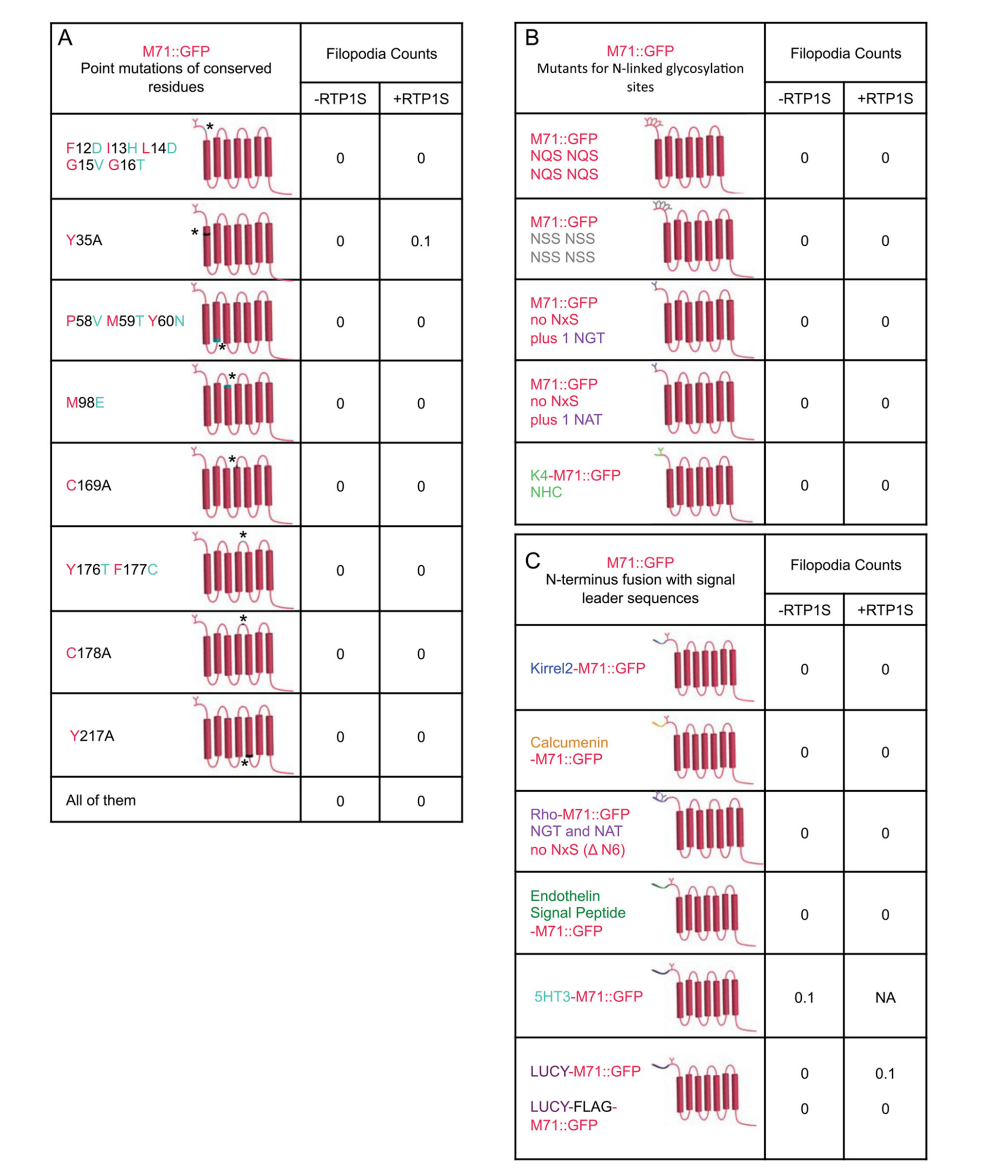
M71::GFP directed mutations that do not traffic to plasma membrane. (A) M71::GFP mutations F12D;I13H;L14D;G15V;G16T (FILGG to DHDGG), Y35A, P58V;M59T;Y60N (PMY to VTN), M98E, C169A, Y176T;F177C (FY to TC), C178A and Y217A. None of these mutations gave raise to any GFP-labeled filopodia. (B) M71::GFP mutations with N-linked glycosylation modifications: 4x NQS, 4x NSS, NQS to NGT, NQS to NAT, Nt to K4 Nt. (C) Addition of leader sequences (Kirrel2, 5HT3, Calcumenin, Rhodopsin, Endothelin, LUCY and LUCY-FLAG) to M71::GFP did not relocate GFP expression into the filopodia. (A-C) Co-expression with RTP1S for all M71::GFP mutations did not increase the presence of any GFP-labeled filopodia.

None of these OR specific sequences appear individually responsible for the ER retention of heterologously expressed M71::GFP mutants as they did not traffic to the filopodia (Fig 4A). To determine if a combination of the homologous sequences were preventing plasma membrane trafficking, we generated a single construct with all of the sequence mutations. There were no observable GFP-labeled filopodia, therefore failure to traffic to the plasma membrane is not due to a combination of these sequences (Fig 4A). Furthermore, co-expression of RTP1S with any mutant did not rescue plasma membrane trafficking (Fig 4A). We were surprised that there was no variation in the non-trafficking phenotype. This led us to believe that other residues might have a stronger influence on trafficking.

It should be noted that the lack of M71::GFP fluorescence in filopodia of OP6 cells is not an intrinsic feature of the assay system. One OR gene that has been very well characterized in a modified HEK293 cell line, is the human OR, OR1A1 [17]. We reasoned that ligands have been identified for this receptor due to some aspect of OR1A1’s amino acid code escapes the classical inability of ORs to traffic in heterologous cell lines (S1A Fig). To confirm our suspicions, we created OR1A1::GFP and expressed it in OP6 cells. GFP-labeled filopodia were identified in many cells (20/20) with an average of 13.7±10.8 (S1B and S1C Fig), which is certainly less robust than mβ2AR. Thus, our assay can detect filopodia for the trafficking of an OR. The amino acid homology of OR1A1 to mβ2AR is only 14% (43/302 residues), which is similar to the 56/302 observed for M71 and mβ2AR (S1A Fig). In addition, the most highly conserved OR superfamily residues in common with mβ2AR are 17/47 for OR1A1, which is similar to the 18/47 for M71, whereas 45/47 residues are conserved with OR1A1 and 46/47 for M71. Thus, OR1A1 and M71 share the same degree of homology with each other compared to the OR superfamily and to mβ2AR, yet OR1A1::GFP does traffic to the plasma membrane and M71::GFP does not. These observations further imply that the highly conserved residues are most likely not involved in retention of M71.

### OR specific N-linked glycosylation properties do not prevent M71 plasma membrane trafficking

N-linked glycosylation at the amino terminus of GPCRs is considered to be a crucial post-translational regulatory step for targeting plasma membrane expression of the protein [18]. The consensus amino acid sequence NxS/T in the amino terminus marks the protein for glycosylation. In ORs, there is usually only one N-linked glycosylation site in the N-terminus (Nt), whereas many other GPCRs contain multiple sites. To test for the role of these sequences on the membrane trafficking of M71::GFP, we generated 5 Nt mutations in M71 (Fig 4B).

M71 has only one N-linked glycosylation site, NQS. We increased the number of NxS/T sequences and assayed for plasma membrane trafficking by adding 4 copies of the OR sequence NQS or 4 copies of the N-linked glycosylation sequence, NSS (as in the mβ2AR) (perhaps better able to be recognized). Neither of these mutations could rescue any observable plasma membrane trafficking.

The addition of 20 amino acids from the Nt of the human Rhodopsin to the Nt of some ORs has been previously reported to improve OR release from the ER. This 20 amino-acid sequence contains two potential glycosylation sites which may contribute to OR plasma membrane trafficking. Replacement of the NQS sequence in M71 with each one of the two N-linked glycosylation sites found in the Nt of the human Rhodopsin, NGT and NAT did not instruct plasma membrane expression. We then added the full Rhodopsin “tag” to the Nt of M71, but it also failed to allow for M71::GFP trafficking to the filopodia (Fig 4C.; see below: addition of signal leader sequence).

Finally, The Nt of the OR K4 contains a non-consensus N-linked glycosylation sequence (NHCT; [19]). Replacement of the first 7 residues of the M71 Nt with those of the K4 OR had no effect on plasma membrane trafficking. We observed the same results when all mutant constructs were co-expressed with RTP1S. These results indicate that the addition of multiple and various N-linked glycosylation sites at the Nt of ORs is not sufficient to restore trafficking to the plasma membrane.

### Addition of signal leader sequences does not rescue M71 membrane trafficking

Since the addition of signal leader tags to GPCRs has provided some success in trafficking varying amounts of receptor protein to the plasma membrane, we analyzed the effect of various signal leader tags on plasma membrane expression of M71. We tested the rescue potential of seven N-terminal signal leader fusion tags: Kirrel2, Calcumenin, 5HT3, Rhodopsin, Endothelin Receptor B, Leucine Rich Repeat Containing 32 (LLRC32)-LUCY, and LUCY plus FLAG. None of the seven tags improved cell surface expression in our assay alone or in the presence of RTP1S (Fig 4C). Several studies showed that RTP1S has a synergistic effect with the addition of Nt tag sequences on ORs. However, in our assay we did not observe such phenomena.

### The Nt and Ct of M71 alone do not prevent plasma membrane expression

**Fig 5:**
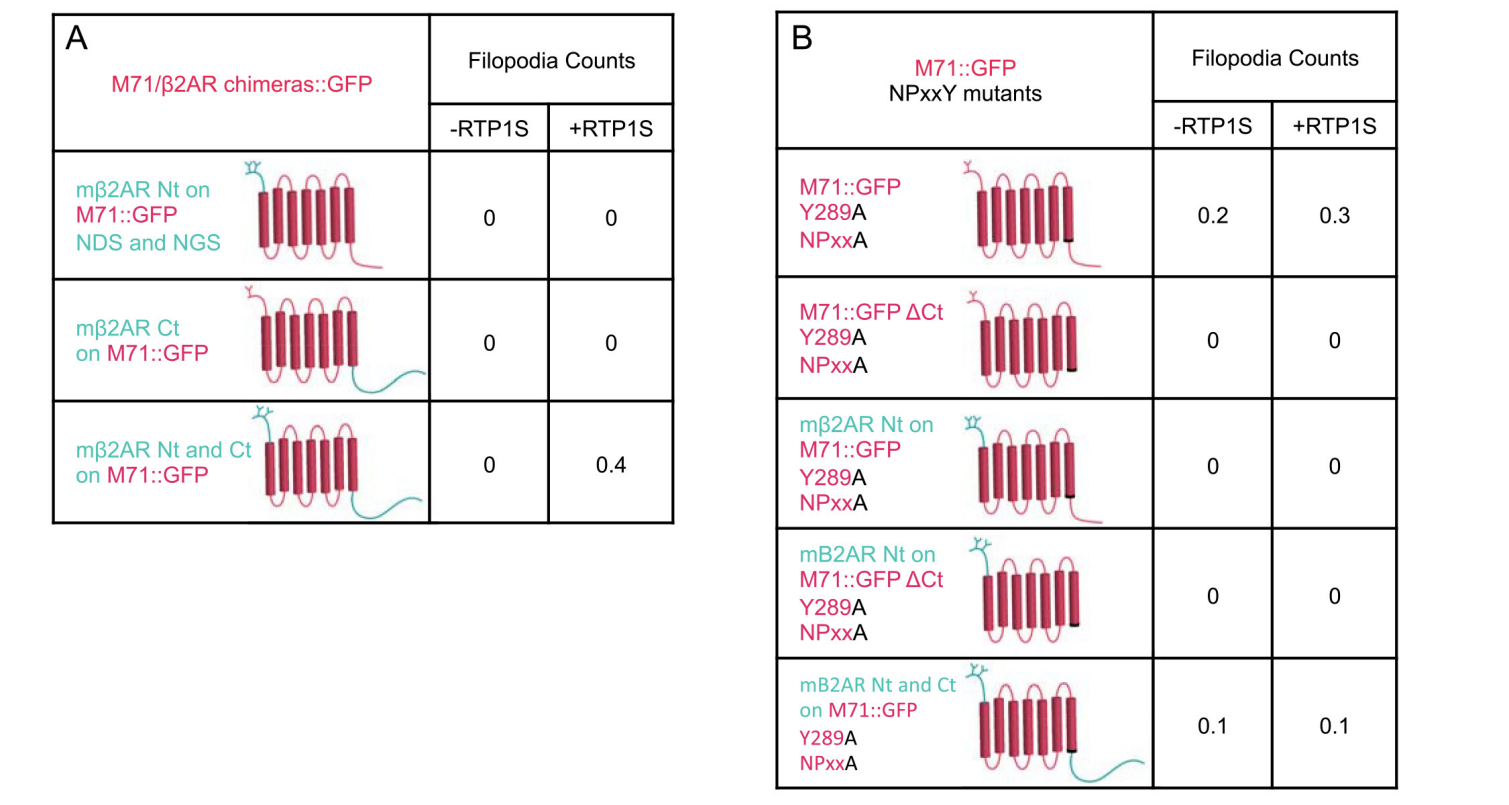
M71::GFP chimeras and truncations that do not traffic to plasma membrane. (A) M71::GFP chimeras with Nt-mβ2AR, Ct-mβ2AR, Nt-and Ct- mβ2AR. None of these chimeras led to GFP-labeled filopodia. (B) Altered NPxxY motif, Y289A M71::GFP mutants, with altered Nt and Ct: WT, ΔCt, Nt-mβ2AR, Nt-mβ2AR/ΔCt, and Nt- and Ct-mβ2AR. None of these chimeras led to GFP labeled filopodia. (A and B) Co-expression with RTP1S for all M71::GFP mutations did not increase the presence of any GFP-labeled filopodia.

We have recently characterized a series of mβ2AR::GFP mutants for their ability to plasma membrane traffic in OP6 cells [9]. Through chimeric analyses with M71, we observed that mβ2AR chimeras with both the Nt and Ct of M71 disrupted plasma membrane trafficking. This synergistic effect appeared intramolecular in origin. Thus, an interaction between the Nt and Ct may be preventing the proper release or folding of ORs in the ER. To test this hypothesis, we replaced the Nt region of M71 with that of mβ2AR so that the region would have structural similarity to that of a trafficking GPCR (Fig 5). We also replaced the C-terminal (Ct) region of M71 with the much longer Ct of mβ2AR. Neither chimera proteins trafficked to the plasma membrane in the presence or in the absence of RTP1S. In addition, swapping both the Nt and Ct of M71 with those of the mβ2AR did not allow for plasma membrane localization (Fig 5A). Therefore, the Nt and the Ct of M71 are not preventing plasma membrane trafficking of its core seven transmembrane domains.

### A Tyrosine 289 mutation (Y289A) within the NPxxY motif of M71 does not rescue membrane trafficking

We have recently reported that the deletion of the Ct (ΔCt) of the mβ2AR blocks its trafficking to the plasma membrane [9]. Surprisingly, the Y326A mutation in a conserved motif at the end of TM7 (NPxxY; [9]) was able to rescue plasma membrane trafficking in the ΔCt mβ2AR. These data may indicate that M71 does not traffic due to Nt and Ct interactions and/or that the M71 Ct behaves like the mβ2AR ΔCt. Thus, we generated the Y289A mutation in the conserved NPxxY motif of M71 in the context of different Nt or Ct or with a ΔCt (Fig 5B). We observed no plasma membrane trafficking of M71 with the Y289A mutation in the context of full-length M71, ΔCt, mβ2AR-Nt, mβ2AR-Nt and ΔCt, or mβ2AR-Nt, -Ct. In addition, the co-expression of RTP1S had no effect on plasma membrane trafficking of those mutants (Fig 5B).

Finally, we tested if ligand activation is possible in absence of plasma membrane expression of our M71::GFP mutants. We expressed several of the previously described M71::GFP mutants in heterologous cells and exposed the cells to 1mM or less with the odor 2-4-dimethyl-acetophenone, which gives the maximum response in M71 expressing OSNs. We did not observe any response in our dose-response assay, performed in HEK 293, for M71::GFP, M71::GFP with Kirrel 2 tag, M71::GFP with LUCY-FLAG tag, M71::GFP with mβ2AR-Nt, -Ct or M71::GFP with mβ2AR-Nt, -Ct and Y289A. We were not surprised by the lack of M71 functionality in heterologous cells, but we were surprised that there was no noticeable improvement of plasma membrane trafficking by site directed mutagenesis, tags or as a chimera with mβ2AR.

### The amino acid composition of odorant receptors is unique amongst GPCRs

Distant members of the OR superfamily can share as little as 18% homology and so a natural conclusion may be that conserved residues amongst ORs are responsible for the observed limited plasma membrane expression in heterologous cells. However, twenty-nine M71 OR mutants did not traffic to the plasma membrane in our *in vitro* structure-function assay. In addition, transfection with OR51E2::GFP, mOR-EG::GFP (olfr73), and mTAAR4::GFP did not show GFP-labeled filopodia, although a few were observed with mOR-EG::GFP when co-transfected with RTP1s (data not shown). These observations indicate that conserved residues may not be responsible for limiting OR expression *in vitro*, so perhaps the non-conserved residues are contributing to the phenomena. To explore this possibility we have taken a comparative bioinformatics approach to determine if any non-conserved residues have a common character in ORs but not amongst other GPCRs and if particular residues are distributed in a pattern in functional domains unique to ORs.

We extracted sequences from the GPCR database for all amine receptors (Amine) and all odorant receptors (ORs) from many species. In addition, we created two subgroups of all amine receptors: all adrenergic receptors (Adrenergic) and all trace amine-associated receptors (TAARs). Both ORs and TAARs are used in the olfactory system to recognize odors. Notably, not all members of the amine receptor superfamily can be functionally expressed *in vitro* in heterologous cells. Within this superfamily, adrenergic receptors are easily expressed *in vitro* whereas TAARs require the rhodopsin 20 amino acid Nt tag and co-expression with OR cofactors (RTP1S/RTP2, REEP1 and Ric8b) [20].

We further refined these sequences to likely functional proteins by excluding pseudogenes with intact coding sequences. The sequences we included must contain two highly conserved motifs: an amino terminal NxS/T glycosylation site and NPxxY motif (resides at the end of transmembrane 7) at distances of 270 to 401 residues. Our final sequences from multiple species for analysis consist of 289 adrenergic receptors, 525 TAARs, 1803 amine receptors, and 12007 odorant receptors (S1 Table).

**Fig 6:**
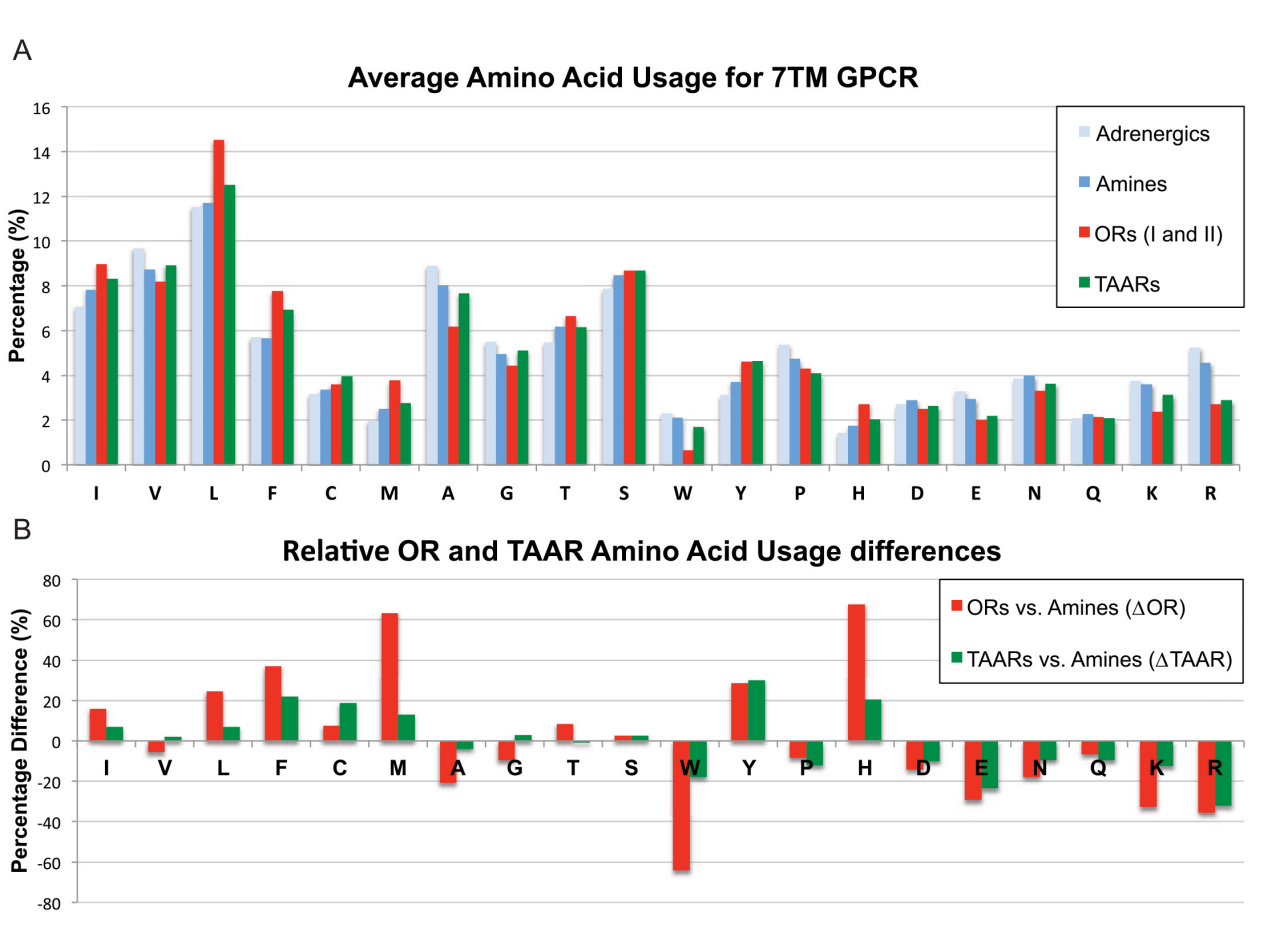
Amino acid residue composition of Adrenergic, Amine, Odorant and Trace amine-associated receptors. (A) Amino acid percentages for Adrenergic, Amine, Odorant (ORs) and Trace amine-associated Receptors (TAARs). ORs and TAARs profiles differ from Adrenergic and Amine receptors. (B) Amino acid differences for ORs and TAARs expressed as percentage difference from Amine receptors. Many residues show an increase in proportion (positive percentage) and decrease in proportion (negative percentage). Three residues for ORs show a large, 60% difference (methionine-M, histidine-H and tryptophan-W). These three residues also show large differences from TAARs with methionine and histidine comprising the bulk of the statistical difference between them. Results did not change if TAARs and Adrenergic receptors were removed from the Amine receptor superfamily. Amino acids are ordered according to their hydropathy index from 4.5 to -4.5 (L to R). It appears that most of the residue differences favor ORs and TAARs to be more hydrophobic.

We then created an amino acid residue profile of all 20 amino acids across these sequences and ranked them by hydropathy index (Fig 6A; [21]). The hydropathy index of an amino acid is a number representing the hydrophobic or hydrophilic properties of its side chains. The larger the number is the more hydrophobic the amino acid. Hydrophobic residues are most commonly found to be internal to a protein, while hydrophilic amino acids tend to be at the surface of the protein. GPCRs contain seven transmembrane domains that directly contribute to their functionality. Not surprisingly, we observed that residues with nonpolar/neutral side chains were highly represented, whereas the residues with polar/charged side chains were poorly represented (Fig 6A). This trend was comparable between the amine and ORs. It should be noted that adding back the rejected sequences did not change our conclusions.

Next, we compared the representation of each residue across ORs and TAARs. We first normalized the percent difference of each residue in the ORs and TAARs to those of the adrenergic receptors, as they successfully traffic to the plasma membrane *in vitro*. We compared the percent difference of each residue between ORs and Amines as well as TAARs vs. Amines. Surprisingly, we saw that both the ORs and TAARs had similar residue composition relative to the Amines with a few notable exceptions: Methionine (M) and Histidine (H) were ~3x more likely to occur in ORs than TAARs and Tryptophan (W) was ~3x less likely to occur in ORs than TAARs (Fig 6B).

### ORs and TAARs have increased hydropathy profiles

GPCRs belong to the 7TM receptor superfamily where each transmembrane segment requires approximately 20 amino acids with an alpha helix and hydrophobic character to pass through the lipid bilayer. Thus, a typical OR or TAAR coding sequence contains approximately 140 residues with a hydrophobic character out of ~320 total residues (~44%). Our analysis of the amino acid usage profile for ORs and TAARs suggested they might have greater hydrophobic character than typical GPCRs. Indeed, we observe that ORs and TAARs relative to amine receptors contain more residues with nonpolar/neutral side chains and fewer residues with polar/charged side chains (Fig 6B).

**Fig 7:**
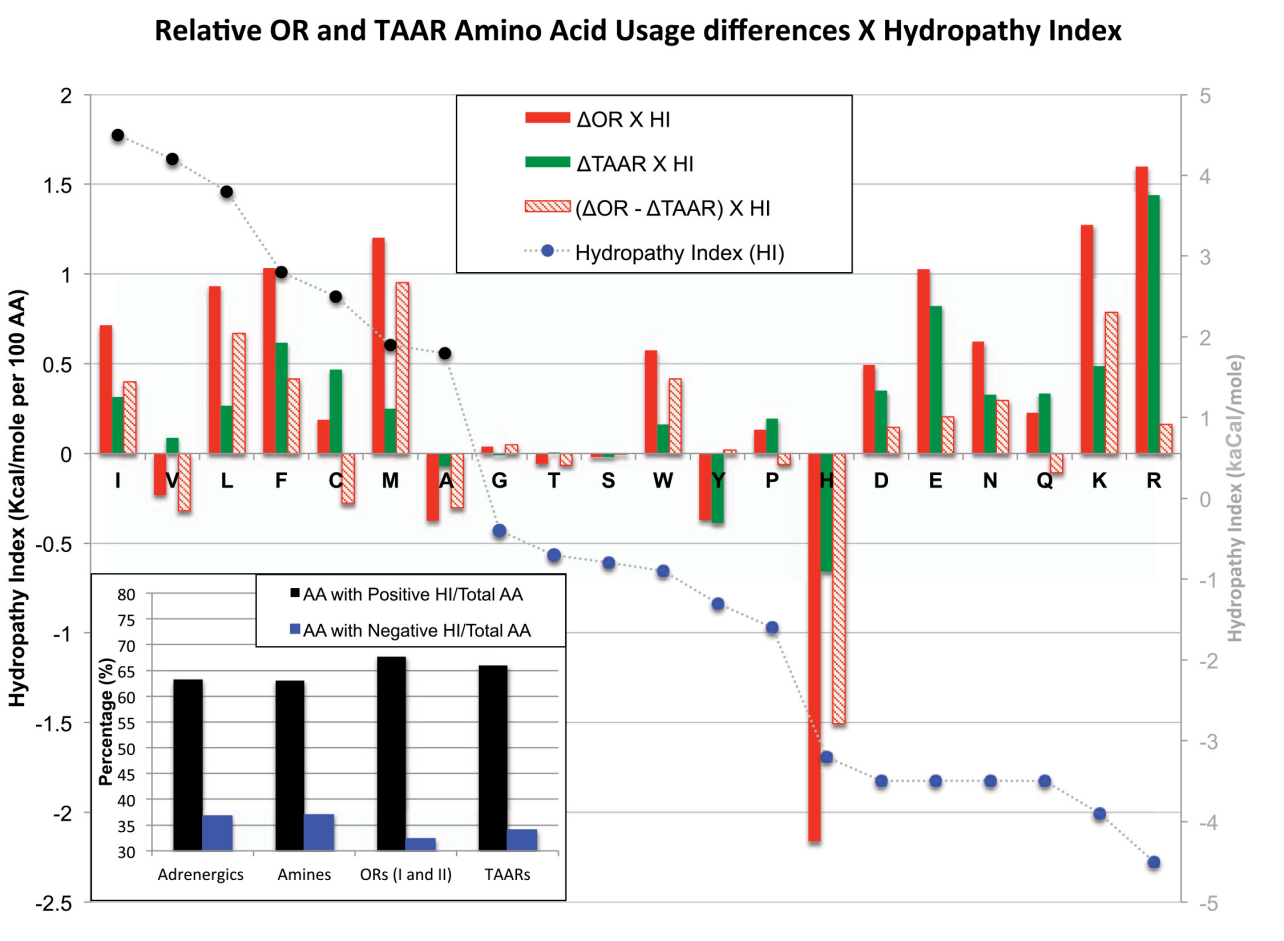
ORs and TAARs have greater hydrophobic character than Adrenergic and Amine receptors. The percent difference in ORs and TAARs compared to Amine receptors was multiplied by the hydropathy index (HI) of each residue. Positive percent differences x positive HI values and negative percent differences x negative HI values increase the hydrophobic character to ORs. Many residues had increased the hydrophobic character or no effect. Histidine showed the most significant difference overall and in relation to TAARs. Amino acids are ordered according to their hydropathy index from 4.5 to -4.5 (L to R). (Inset) The positive and negative hydrophobic character of Adrenergic, Amine, ORs and TAARs wre calculated by adding together all residues that contribute to non-polar/neutral or polar/positive-negative hydropathy index and calculating the percentage relative to the total residues. There was a greater percentage of positive hydropathy and reduced negative hydropathy values for ORs and TAARs compared to Adrenergic and Amine receptors. This translated into higher ratios of polar/neutral to polar/positive-negative residues.

The Hydropathy index (HI) assigns values from -4.5 to 4.5 for each amino acid [21] where a value of 4.5 indicates that amino acid is most likely to be found in a transmembrane domain. When we compare the residues with negative hydropathy indexes (single residue codes: G, T, S, W, Y, P, H, D, E, N, Q) versus positive hydropathy indexes (single residue codes: I, V, L, F, C, M, A), an overall 4% decrease in negative HI values and an overall 4% increase in positive HI values for ORs relative to both Adrenergic and Amine receptors were observed (Fig 7, inset). By contrast only a 2% change in the high and low HI values for TAARs is found compared to the amine receptor superfamily. These changes translate into a net increase in twelve hydrophobic residues for an OR and six for a TAAR coding region. However, the percentage of total positive and negative HI-residues for Adrenergic receptors were nearly identical to the Amine receptor superfamily. To determine how the increase or decrease in HI observed for each residue translated into increased hydropathy of ORs we multiplied the percent difference of each amino acid with its associated HI value. A positive value indicates that this residue change would promote the hydropathy of a protein (positive percent residue difference x positive HI value and negative percent residue difference x negative HI value) (Fig 7).

We find that all residues enriched in ORs and TAARs except for histidine either have no effect or promote hydropathy for both the ORs and TAARs (Fig 7). The gain of isoleucine (I), leucine (L), phenylalanine (F), and methionine (M) residues and loss of tryptophan (W), glutamic acid (E), asparagine (N), lysine (K), arginine (R) residues promoted hydropathy for both ORs and TAARs, but nearly all were substantially higher in ORs (Fig 7). In TAARs, only glutamic acid (E) and arginine (R) are similarly fewer in percentage compared to ORs. However, we observe a 60% increase in histidine (H) residues for ORs, which translated into the largest net negative hydropathy, values for ORs and was 75% greater than TAARs (Fig 7). Thus, all residues enriched in ORs and TAARs impart greater hydropathy values with the exception of histidine (H) in ORs, and suggest that histidine (H) likely provides ORs with a specific function that cannot be substituted by another amino acids.

#### Distribution profiling of Histidine, Methionine and Tryptophan residues

**Fig 8:**
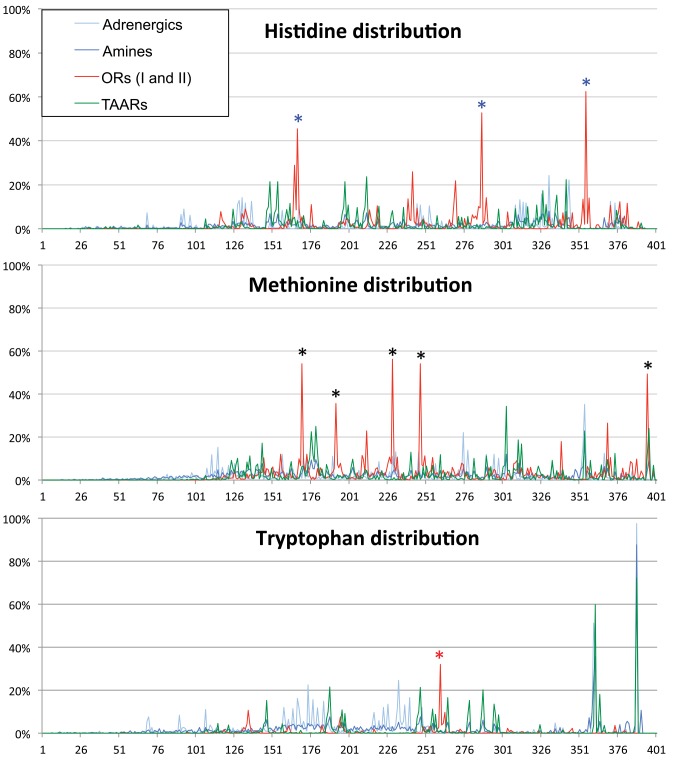
Distribution of Methionine, Histidine and Tryptophan in Adrenergic, Amine, OR, TAAR receptors. (A) Distribution of histidine residues; (B) Distribution of methionine residues; and (C) Distribution of tryptophan residues in Adrenergic, Amine, Odorant, and Trace amine-associted receptors. The frequency of occurrence is displayed from NxS/T to NPxxY conserved motifs, however the graphs are all anchored by the Ct NPxxY motif. Three histidine and five methionine residues are found at discrete regions in ORs compared all other receptor types (blue and black asterisks); See Fig 3 for exact position. Tryptophan residues are nearly abolished in ORs with only one conserved position (red asterisk), but distributions are conserved between TAARs and Adrenergic receptors.

Our analyses of amino acid usage have revealed greater representation of histidine (H) and methionine (M) residues and lower representation of tryptophan (W) residues in ORs and TAARs compared to Amine receptors. The functional implications of this difference may differ if the distribution of H, M, and W residues were broadly distributed throughout the protein versus localized to particular protein domains (Fig 8; S2, S3, S4 and S5 Figs).

We analyzed the distribution of H and M residues for Adrenergic, Amine, ORs and TAARs and reveal broad distribution patterns. Interestingly, within these broad distribution patterns, we see specific localization of some of these residues at particular sites in ORs. There are three notable exceptions for H residues and five for M residues with all eight being highly conserved (Fig 3). Interestingly, 7/8 peaks correspond to conserved sequence motifs found in predicted intracellular (IC), extracellular (EC) or transmembrane (TM) portions of the ORs: L**H**TP**M**Y (IC1), PK**M**L (EC1), **M**AYDRYVAIC (end of TM3), **H**FFCD (EC2), CSS**H** (IC3), P**M**LNPLIY (end of TM7). The role of the EC motif PK**M**L is unknown, but **H**FFCD may bind metal ions. The IC located motifs: **M**AYDRYVAIC, CSS**H** and P**M**LNPLIY are likely involved in signaling events. The intracellular portion L**H**TP**M**Y has no known function, but when P**M**Y is mutated to VTN in M71::GFP (Fig 4), we observe no change in plasma membrane trafficking in heterologous cells.

Finally and very surprisingly, we observe that W residues are nearly absent in ORs. For Adrenergic receptors, Amine receptors and TAARs, W residues are found at the Ct, and for Adrenergic receptors and TAARs, W residues are also found within two internal clusters. The rare W residue in ORs is within TM4, which is a highly conserved position within TM4 across several superfamilies: ORs, TAARs, Vomeronasal receptors (VRs), Adrenergic families (Figs 3 and 8; S2, S3, S4 and S5 Figs). We also observe dispersed peaks of W residues in Adrenergic receptors and TAARs within the middle portion of the protein. This observation reveals a lack of sequence length conservation between these receptor groups whereas sequence length conservation is maintained in ORs (95% of ORs have the same length of ~286 residues from NxS/T/C to NPxxY). In conclusion, only a single W residue appears weakly conserved amongst ORs compared to conservation of M and H residues, and no W residues are observed in the predicted M71 protein (Figs 3 and 8C).

## Discussion

To efficiently characterize and identify ligands, GPCRs have been expressed in heterologous cell lines. Many of the earliest identified GPCRs including Rhodopsin and β2 adrenergic receptor (β2AR) robustly express in culture, which has made their detailed characterization possible.

Since the identification of ORs twenty-five years ago there has been persistent effort to express them in heterologous cell lines and identify odors or ligands they are capable of binding [22]. OR expression in cells is typically poor with most protein localized to the ER or intracellular compartments and absent at the plasma membrane [23]. Several laboratories have identified protein cofactors (RTP1S, RTP2, REEP1, Ric8b) that rescue some OR protein expression at the plasma membrane when co-expressed with ORs, albeit at extremely low levels compared to the overall protein expression. However, these assays require the OR to be tagged at the amino terminus tags for plasma membrane expression. These tags range from HA, FLAG, Rho or the recently identified LUCY or LUCY-FLAG. Co-expression of tagged ORs along with several cofactors can confer some plasma membrane in several heterologous cell lines including HEK293T and its derivative HANA3A.

These tools for limited plasma membrane expression do not work with all ORs. The M71 OR is one such receptor that is refractory to *in vitro* expression and targeting to the plasma membrane. However, the *in vivo* functionality of the M71 OR in OSNs has been described in depth with regards to genetic and physiological mechanisms and its protein localization utilizing immunohistochemistry.

### *In vivo* characterization of Ct-tagged OR M71 with GFP

To better model and describe M71 protein distribution and function in live olfactory neurons we tagged the Ct of the protein with GFP. We have successfully used this method for analyzing the expression of an other GPCR, the β2AR [10]. We previously generated gene-targeting mice expressing the M71::GFP fusion to define M71 protein localization. We detected GFP in both olfactory cilia and surprisingly olfactory axons during the process of glomerular formation in the olfactory bulb [11]. M71 specific antibodies substantiated both results [24]. In the olfactory system, interactions between like axons lead to their coalescence into specific glomeruli. It is the OR sequence that imparts this axonal identity to these axons. The homogeneous glomeruli formed from M71::GFP expressing axons located close to the M71 glomeruli, as posterior but more dorsal. Oddly, M71::GFP glomeruli and M71 glomeruli were distinct; axons of each group did not coalesce with the other OR identity. One possibility for this differential axonal identity and glomerular formation could be from M71::GFP having reduced or modified expression or function. We have already generated a second M71 mutation, which was engineered to express less M71 (IRES-M71), which formed glomeruli 1.5mm anterior and ventral to the normal M71 and M71::GFP glomeruli. OSNs expressing IRES-M71 retain identical functionality of M71 to the high affinity agonist 2aACP despite a 3x reduction in protein in the olfactory cilia. But, the lower affinity agonist 4mACP was altered by one log EC50 in IRES-M71 neurons [14].

Here, we have functionally characterized the M71::GFP expressing OSNs with 7 odors: five that have robust affinity for M71 and two that have high affinity for the OR M72, which shares 96% amino acid identity with M71. All five odors that activate M71 also activate M71::GFP whereas both odors that activate M72 fail to activate M71::GFP. However, we find that odorant 4mACP has one log higher EC50 to M71::GFP comparable to the IRES-M71 profile. Thus, on first approximation M71::GFP is phenotypically expressed at the same level as IRES-M71, which is 3x lower than M71. It is likely that either M71::GFP and IRES-M71 protein is expressed at the same level or M71::GFP has 3x less functional protein at the membrane. It seems unlikely that G-protein coupling was globally affected, as the dose response profiles of the higher affinity agonists were the same.

The characterization of IRES-M71 neurons with all 7 agonists reveals a slightly broadened response and includes the M72 specific ligand BTP (Fig 1), which was not observed for M71::GFP neurons. So why does IRES-M71 have a different response to BTP or perhaps more importantly, does IRES-M71 differ functionally from M71::GFP or M71? When M71 is expressed from an IRES sequence, translation is initiated at the start codon within the IRES, but there is an upstream in-frame start codon that may add four additional amino acids (MATT). We have recently shown that Nt and Ct interactions affect OR trafficking and Ct mutations affect functionality, thus it is possible that the addition of the 4 amino acids, MATT, might alter G-protein coupling efficiencies and lead to the slight BTP excitation [9].

There are four clear conclusions from the *in vivo* analysis of M71::GFP. First, addition of GFP to the Ct has minimal affects on the signaling capacity of M71 or the position of glomerular formation as the M71::GFP glomeruli is in close proximity to the M71 glomeruli. Second, we did not observe a lower maximum response for M71::GFP using any of the odors, but we did observe one log changes in EC50 for 4mACP. This is in contrast to our previous study regarding the mβ2AR::GFP revealed a lower maximum response rate [10], which suggests a change in level of functional protein expression. Third, the mβ2AR::GFP may have differential response profiles from mβ2AR should a broader ligand range be tested. Finally, if the potential for odor-evoked activity of the OR were a significant feature of positional shifts in glomerular formation, then the position of glomerular formation for M71::GFP and IRES-M71 should have been the same. In this regard, M71 and M72 project to similar locations in the bulb despite the M72 odor-evoked profile being significantly different from M71 and IRES-M71, which both have very similar odor-evoked profiles. Thus, the most likely cause of IRES-M71 glomerular formation at such a different location from M71, M71::GFP or M72 is due to a change in its axonal identity code (alteration of its 309 residues) such as the addition of four residues in the Nt or an unanticipated truncation. To translate these differences into a 1.5mm shift in glomerular formation suggests that OR multimers might be involved to amplify what appears to be a minor modification of the protein.

### M71::GFP does not traffic to the plasma membrane in heterologous cells

We have successfully described the localization of mutant mβ2AR protein using Ct GFP fusions in OP6 cells and have implemented this tool to characterize M71 signaling properties *in vitro* and *in vivo* (OSNs). The Ct GFP modification does not appear to interfere with receptor trafficking *in vivo* and only slightly alters the signaling efficacy *ex vivo* of our tested M71 specific odors.

To determine if there are amino acids within the ORs that prevent functional plasma membrane expression *in vitro*, we made a series of M71 mutants visualized with the Ct GFP tag. These mutations targeted conserved residues amongst ORs, conserved residues not known to be involved in signal transduction, N-linked glycosylation additions, Nt tags, and chimeric receptors with mβ2AR (Figs **4**and **5**). None of these mutations or a combination of these mutations altered plasma membrane trafficking even when co-expressed with RTP1S. Although we were unable to identify a modifiable sequence to restore plasma membrane trafficking, we did set up an assay for potential rapid screening of OR mutants derived by random mutagenesis.

Initially we made subtle mutations to M71 that might not affect G-protein coupling, but when those failed to affect trafficking, we generated mutations that were more likely to affect the overall structure of the protein and coupling efficiencies. When larger domain swaps of M71 did not affect trafficking, we concluded that there might be a broad feature of ORs that was preventing functional expression in heterologous cells.

Why does robust functional expression of ORs appears limited to OSNs in the olfactory system where they act to identify odors in the nasal mucosa? Two simple possibilities emerge: First, OSNs express specific cofactors such as RTP1S that might help shepherd ORs to the plasma membrane, but RTP1S does not help all ORs including M71 traffic to the plasma membrane in heterologous cells. This indicates that the presence of an olfactory specific cofactor is not necessarily sufficient to rescue the trafficking deficit. Perhaps multiple cofactors are needed. Second, the lipid composition of OSNs could be unique and it is this environment that permits OR insertion into the plasma membrane. It is possible that ORs cannot traffic in heterologous cells as a result of a combination of necessary cofactors, lipid modifications, and the hydrophobic character, which we report, that all contribute to successful OR plasma membrane trafficking in OSNs. The need for OSN specific cofactors or lipids could be tested in OSNs cultured *in vitro* and forced to overexpress an OR.

### OSN expressed ORs and TAARs are enriched in hydrophobic character

There is very limited homology amongst distant members of the OR superfamily as revealed by their amino acid sequence alignment. But, homology is found amongst highly conserved residues, many of which are conserved in GPCRs outside of ORs. Thus, if any single conserved residue across ORs were contributing to the suppression of plasma membrane trafficking, our extensive mutagenesis might have identified it. By inference, if the OR conserved residues are not contributing to the inhibition of OR trafficking in heterologous cells, then it is the non-conserved residues that are likely playing a role in plasma membrane trafficking.

We took a comparative bioinformatics approach to determine if the protein properties of ORs and TAARs differed from Adrenergic and Amine receptors. The Nt and Ct of ORs and TAARs are usually short, whereas the Nt and Ct of Amine and Adrenergic receptors are usually longer. We confronted this difference in our mutagenesis analysis and determined that the length of the Nt and Ct were not contributing to plasma membrane trafficking. We then asked if the core sequences between the amino terminal N-linked glycosylation sites and TM7 motif, NPxxY differed in hydrophobic character among the superfamilies.

Our *in silico* analysis revealed that 19/20 amino acid residues either had no affect on or increased the hydrophobic character of ORs and TAARs. Interestingly, despite a negative HI, histidine has the single greatest contribution of a residue to the overall hydrophobic character of an OR. But, the negative HI of histidine was dwarfed by the total hydropathy contribution of the other 19 residues. Methionine residues are also more prevalent in ORs compared to Adrenergic and Amine receptors and we speculate that it may act to provide a balance to the hydrophilic addition of histidine in the tertiary structure. Both residues appear in highly conserved motifs, but only appear together in one of them, L**H**TP**M**Y (Fig 3). None of the histidine residues (0/3) and only two methionine residues (2/5) are found in transmembrane regions (Fig 3). It is possible that these residues may contribute directly to the tertiary structure of the OR in order to compensate for its overall enriched hydrophobic character. Mutations of 5/8 conserved methionine and histidine residues abolish or reduce the function of MOR244-3; Mutations of H56K and M59A from L**H**NP**M**Y, M81A from PK**M**L and M118A from **M**AYDRYVAIC reduce activity, while H243 mutants from CSA**H** completely abolish activity and trafficking. Methionine and histidine have been postulated in the binding transition metals such as copper [25] and could thus contribute to the stability of the OR protein or to the ability of the OR to bind odorants.

The unique amino acid composition of ORs likely contributes to their functionality, and modifying this functionality for protein expression *in vitro* can yield protein that may no longer reflect *in vivo* structure or function.

### Physiological functions of odorant receptors

The unique amino acid usage of ORs we reveal in this study might have several implications for their functionality. The organization of the olfactory system is set up such that an organism cannot detect any odorant information if glomerular formation has not occurred. Thus, it is more critical to olfactory function that ORs traffic to the plasma membrane where they can contribute to axonal identity and glomerular formation rather than bind an odor with high-affinity. In this scenario an OR with high-affinity for a given odor may not necessarily be able to impart axon identity and so its OSNs never contribute to the coding of that olfactory stimulus. By contrast, if glomerular formation occurs for axons that express an OR with weak binding affinity to an odor(s), then that OR can still contribute to stimulus detection. Thus, amino acid usage may have more to do with the role of a specific OR in axon guidance than in ligand detection.

It is also possible that plasma membrane targeting of ORs is linked to its role in stabilizing the expression of a chosen OR allele during the mechanism of singular gene choice. In OSNs, this process is critical for OR expression as robust co-expression of a 2^nd^ OR has not been observed. In heterologous cells, the absence of a mechanism of OR gene choice may be why ORs also poorly traffic to the plasma membrane. If so, robust plasma membrane trafficking in heterologous cells can only occur if those cells are tricked into “choosing” and/or “stabilizing” an OR for expression.

## Conclusion

We were surprised that our extensive mutational analysis of OR M71 failed to even partially improve its membrane trafficking in our assay. We then realized that the nature of the OR sequence might be fundamentally different from other 7TMs based on our *in silico* analysis of the OR superfamily. Going forward, it may be difficult to readily determine a rule generalizable to all ORs as to why many ORs fail to reach the plasma membrane, but allow some ORs to partially traffic, such as OR1A1. Using both our assay and random mutagenesis of an OR that partially traffics to the plasma membrane, we may be able to identify a set of mutations that help decipher this seemingly intractable code.

## Supporting Information

S1 Fig Conserved OR sequences revealed by logo plot shared with M71, OR1A1 and mβ2AR. (A) OR logo using mouse Class I and II odorant receptor sequences. Most highly conserved OR residues are depicted with black circles. M71, OR1A1 and mβ2AR sequences are delineated underneath logo plot; Residues with greater than 50% bit conservation to OR logo are underlined. M71 residue swaps between M71 and mβ2AR are delineated in purple and green, respectively. M71 residues converted to alanine are described with bold blue A. Conserved residues between M71, OR1A1 and mβ2AR only are in bold and maroon. Conserved residues between M71, OR1A1 and not mβ2AR are in bold and orange. Conserved residues between OR1A1, mβ2AR and not M71 are in bold and light blue. Conserved residues between M71, mβ2AR and not OR1A1 are in bold and red. Five conserved methionine residues, three conserved histidine residues and one weakly conserved tryptophan residue marked with asterisk. Two of these residues are also found in OSN expressed TAARs marked with red asterisk. (B, C) Two examples of OR1A1::GFP transfected OP6 cells. GFP-labeled filopodia were readily observed (arrowhead).
(EPS)

S2 Fig Distribution of histidine, methionine and tryptophan residues in Adrenergic receptors. The frequency of occurrence is displayed from NxS/T to NPxxY conserved motifs, however the graphs are all anchored by the Ct NPxxY motif.
(EPS)

S3 Fig Distribution of histidine, methionine and tryptophan residues in Amine receptors. The frequency of occurrence is displayed from NxS/T to NPxxY conserved motifs, however the graphs are all anchored by the Ct NPxxY motif.
(EPS)

S4 Fig Distribution of histidine, methionine and tryptophan residues in OR (I and II) receptors. The frequency of occurrence is displayed from NxS/T to NPxxY conserved motifs, however the graphs are all anchored by the Ct NPxxY motif. Three histidine and five methionine residues are found at discrete regions in ORs compared all other receptor types (blue and black asterisks); See Fig 3 for exact position. Tryptophan residues are nearly abolished in ORs with only one conserved position (red asterisk), but distributions are conserved between TAARs and Adrenergic receptors.
(EPS)

S5 Fig Distribution of histidine, methionine and tryptophan residues in TAAR receptors. The frequency of occurrence is displayed from NxS/T to NPxxY conserved motifs, however the graphs are all anchored by the Ct NPxxY motif.
(EPS)

S1 File Accepted and Rejected GPCR sequences. (ZIP)

S1 Table Raw data, amino acid content per each GPCR group. (XLSX)

S1 Text Nucleotide and amino acid sequences used in mutational analysis and their plasmid names. (DOCX)
