# Supplementary material for: In Vitro Mutational and Bioinformatics Analysis of the M71 Odorant Receptor and Its Superfamily
Source: PLoS One. 2015 Oct 29;10(10):e0141712. doi: 10.1371/journal.pone.0141712 (PMC4626375; doi:10.1371/journal.pone.0141712)
Supplement: S1 Text — (DOCX) [file pone.0141712.s008.docx]

# S1 Text. Nucleotide and amino acid sequences used in mutational analysis

**GFP with linker used for all fusion constructs:**

CGATCCACCGGTCGCCACCatggtgagcaagggcgaggagctgttcaccggggtggtgcccatcctggtcgagctggacggcgacgtaaacggccacaagttcagcgtgtccggcgagggcgagggcgatgccacctacggcaagctgaccctgaagttcatctgcaccaccggcaagctgcccgtgccctggcccaccctcgtgaccaccctgacctacggcgtgcagtgcttcagccgctaccccgaccacatgaagcagcacgacttcttcaagtccgccatgcccgaaggctacgtccaggagcgcaccatcttcttcaaggacgacggcaactacaagacccgcgccgaggtgaagttcgagggcgacaccctggtgaaccgcatcgagctgaagggcatcgacttcaaggaggacggcaacatcctggggcacaagctggagtacaactacaacagccacaacgtctatatcatggccgacaagcagaagaacggcatcaaggtgaacttcaagatccgccacaacatcgaggacggcagcgtgcagctcgccgaccactaccagcagaacacccccatcggcgacggccccgtgctgctgcccgacaaccactacctgagcacccagtccgccctgagcaaagaccccaacgagaagcgcgatcacatggtcctgctggagttcgtgaccgccgccgggatcactctcggcatggacgagctgtacaagtaa

**D357: mB2AR**

ATGGGGCCACACGGGAACGACAGCGACTTCTTGCTGGCACCCAACGGAAGCCGAGCGCCAGACCACGACGTCACTCAGGAACGGGACGAAGCGTGGGTTGTGGGCATGGCCATCCTCATGTCGGTTATCGTCCTGGCCATCGTGTTTGGCAACGTGCTGGTCATCACGGCCATTGCCAAGTTCGAGCGACTACAAACCGTCACCAACTACTTCATAATCTCCTTGGCGTGTGCTGATCTAGTCATGGGCCTAGCGGTGGTGCCGTTTGGGGCCAGTCACATCCTTATGAAAATGTGGAATTTTGGCAACTTCTGGTGCGAGTTCTGGACTTCCATTGATGTGTTGTGCGTCACAGCCAGCATCGAGACCCTGTGCGTGATTGCAGTGGATCGCTATGTTGCTATCACATCGCCCTTCAAGTACCAGAGCCTGCTGACCAAGAATAAGGCCCGAGTGGTCATCCTGATGGTATGGATTGTATCTGGCCTTACCTCCTTTTTGCCTATCCAGATGCACTGGTACCGTGCCACCCACAAGAAAGCTATCGATTGTTACACCGAGGAGACTTGCTGTGACTTCTTCACGAACCAGGCCTACGCCATCGCGTCCTCGATTGTGTCTTTCTACGTGCCCCTGGTGGTGATGGTCTTTGTCTATTCCCGGGTCTTCCAGGTGGCCAAAAGGCAGCTGCAGAAGATAGACAAATCTGAAGGAAGATTCCACGCCCAAAACCTCAGCCAGGTGGAGCAGGATGGGCGGAGCGGCCACGGACTCCGAAGGTCCTCCAAGTTCTGCTTGAAAGAGCACAAAGCCCTCAAGACTTTAGGCATCATCATGGGCACATTCACCCTCTGCTGGCTGCCCTTCTTCATTGTCAATATCGTGCACGTTATCAGGGACAACCTCATCCCTAAGGAAGTTTACATTCTCCTTAACTGGTTGGGCTACGTCAACTCTGCCTTCAATCCTCTTATCTACTGTCGGAGTCCAGATTTCAGGATTGCCTTTCAAGAGCTTCTGTGCCTTCGCAGGTCTTCTTCGAAAACCTATGGGAACGGCTACTCTAGCAATAGCAACGGCAGAACGGACTACACAGGGGAGCCAAACACTTGTCAGCTGG

GGCAGGAGAGAGAACAGGAACTGCTGTGTGAGGATCCCCCAGGCATGGAAGGCTTTGTGAACTGTCAAGGTACTGTGCCTAGCCTTAGCGTTGACTCCCAAGGAAGGAACTGTAGTACAAATGACTCGCCACTGTTAATTAA

**D1569-1: RTP1S**

atgtgtaagagtgtgaccacaggtgagtggaagaaggtcttctacgagaagatggaggaggtgaagccagcggacagctgggacttcatcatagaccccaacctcaagcacaatgtgttggcccctggctggaagcagtacctggaacttcatgcctcaggcaggttccactgttcctggtgctggcacacctggcagtcaccccatgtagtcatcctcttccacatgtacctggacaaggctcagcgcgctggttcggtgcgcatgcgtgtgttcaagcagctctgctacgagtgcggtacagcacggctggatgagtccagcatgctggaggagaacatcgaaagcctggtggacaacctcatcaccagtttgcgagagcagtgctacggggagcgtggtggccactaccgcatccatgtggccagccggcaggacaaccggcgacaccgcggagagttctgcgaggcctgccaggaaggcatcgtgcactggaagcccagtgagaagctgctggaggaggaggcgaccacctacaccttctcccgtgctcccagccccaccaaaccgcaggctgaaacaggctcaggctgcaacttctgctccattccctggtgcttattttgggccacggttttgatgctcatcatctacctgcaattctccttccgtacttctgtctaa

**D358: M71**

ATGACCGCCGAGAACCAGAGCACCGTGACCGAGTTCATCCTGGGCGGCCTGACCAACAGACCCGAGCTGCAGCTGCCCCTGTTCCTGCTGTTCCTGGGCATCTACGTGGTGACCATGGTGGGCAACCTGGGCATGATCACCCTGATCGGCCTGAACAGCCAGCTGCACACCCCCATGTACTTCTTCCTGAGCAACCTGAGCCTGGTGGACCTGTGCTACAGCAGCGTGATCACCCCCAAGATGCTGATCAACTTCGTGAGCCAGAGAAACCTGATCAGCTACGTGGGCTGCATGAGCCAGCTGTACTTCTTCCTGGTGTTCGTGATCGCCGAGTGCTACATGCTGACCGTGATGGCCTACGACAGATACGTGGCCATCTGCCAGCCCCTGCTGTACAACATCATCATGAGCCCCGCCCTGTGCAGCCTGCTGGTGGCCTTCGTGTACGCCGTGGGCCTGATCGGCAGCGCCATCGAGACCGGCCTGATGCTGAAGCTGAACTACTGCGAGGACCTGATCAGCCACTACTTCTGCGACATCCTGCCCCTGATGAAGCTGAGCTGCAGCAGCACCTACGACGTGGAGATGGCCGTGTTCTTCCTGGCCGGCTTCGACATCATCGTGACCAGCCTGACCGTGCTGATCAGCTACGCCTTCATCCTGAGCAGCATCCTGAGAATCAGCAGCAACGAGGGCAGAAGCAAGGCCTTCAGCACCTGCAGCAGCCACTTCGCCGCCGTGGGCCTGTTCTACGGCAGCACCGCCTTCATGTACCTGAAGCCCAGCACCGCCAGCAGCCTGGCCCAGGAGAACGTGGCCAGCGTGTTCTACACCACCGTGATCCCCATGTTCAACCCCCTGATCTACAGCCTGAGAAACAAGGAGGTGAAGACCGCCCTGGACAAGACCCTGAGAAGAAAGGTGTTCTTAATTAA

**D371: M71 F12D I13H L14D G15V G16T**

ATGACCGCCGAGAACCAGAGCACCGTGACCGAGgaccacgacgtGacCCTGACCAACAGACCCGAGCTGCAGCTGCCCCTGTTCCTGCTGTTCCTGGGCATCTACGTGGTGACCATGGTGGGCAACCTGGGCATGATCACCCTGATCGGCCTGAACAGCCAGCTGCACACCCCCATGTACTTCTTCCTGAGCAACCTGAGCCTGGTGGACCTGTGCTACAGCAGCGTGATCACCCCCAAGATGCTGATCAACTTCGTGAGCCAGAGAAACCTGATCAGCTACGTGGGCTGCATGAGCCAGCTGTACTTCTTCCTGGTGTTCGTGATCGCCGAGTGCTACATGCTGACCGTGATGGCCTACGACAGATACGTGGCCATCTGCCAGCCCCTGCTGTACAACATCATCATGAGCCCCGCCCTGTGCAGCCTGCTGGTGGCCTTCGTGTACGCCGTGGGCCTGATCGGCAGCGCCATCGAGACCGGCCTGATGCTGAAGCTGAACTACTGCGAGGACCTGATCAGCCACTACTTCTGCGACATCCTGCCCCTGATGAAGCTGAGCTGCAGCAGCACCTACGACGTGGAGATGGCCGTGTTCTTCCTGGCCGGCTTCGACATCATCGTGACCAGCCTGACCGTGCTGATCAGCTACGCCTTCATCCTGAGCAGCATCCTGAGAATCAGCAGCAACGAGGGCAGAAGCAAGGCCTTCAGCACCTGCAGCAGCCACTTCGCCGCCGTGGGCCTGTTCTACGGCAGCACCGCCTTCATGTACCTGAAGCCCAGCACCGCCAGCAGCCTGGCCCAGGAGAACGTGGCCAGCGTGTTCTACACCACCGTGATCCCCATGTTCAACCCCCTGATCTACAGCCTGAGAAACAAGGAGGTGAAGACCGCCCTGGACAAGACCCTGAGAAGAAAGGTGTTCTGA

**D372: M71 Y35A**

ATGACCGCCGAGAACCAGAGCACCGTGACCGAGTTCATCCTGGGCGGCCTGACCAACAGACCCGAGCTGCAGCTGCCCCTGTTCCTGCTGTTCCTGGGCATCgcCGTGGTGACCATGGTGGGCAACCTGGGCATGATCACCCTGATCGGCCTGAACAGCCAGCTGCACACCCCCATGTACTTCTTCCTGAGCAACCTGAGCCTGGTGGACCTGTGCTACAGCAGCGTGATCACCCCCAAGATGCTGATCAACTTCGTGAGCCAGAGAAACCTGATCAGCTACGTGGGCTGCATGAGCCAGCTGTACTTCTTCCTGGTGTTCGTGATCGCCGAGTGCTACATGCTGACCGTGATGGCCTACGACAGATACGTGGCCATCTGCCAGCCCCTGCTGTACAACATCATCATGAGCCCCGCCCTGTGCAGCCTGCTGGTGGCCTTCGTGTACGCCGTGGGCCTGATCGGCAGCGCCATCGAGACCGGCCTGATGCTGAAGCTGAACTACTGCGAGGACCTGATCAGCCACTACTTCTGCGACATCCTGCCCCTGATGAAGCTGAGCTGCAGCAGCACCTACGACGTGGAGATGGCCGTGTTCTTCCTGGCCGGCTTCGACATCATCGTGACCAGCCTGACCGTGCTGATCAGCTACGCCTTCATCCTGAGCAGCATCCTGAGAATCAGCAGCAACGAGGGCAGAAGCAAGGCCTTCAGCACCTGCAGCAGCCACTTCGCCGCCGTGGGCCTGTTCTACGGCAGCACCGCCTTCATGTACCTGAAGCCCAGCACCGCCAGCAGCCTGGCCCAGGAGAACGTGGCCAGCGTGTTCTACACCACCGTGATCCCCATGTTCAACCCCCTGATCTACAGCCTGAGAAACAAGGAGGTGAAGACCGCCCTGGACAAGACCCTGAGAAGAAAGGTGTTCTGA

**D374: M71 PMY to VTN**

ATGACCGCCGAGAACCAGAGCACCGTGACCGAGTTCATCCTGGGCGGCCTGACCAACAGACCCGAGCTGCAGCTGCCCCTGTTCCTGCTGTTCCTGGGCATCTACGTGGTGACCATGGTGGGCAACCTGGGCATGATCACCCTGATCGGCCTGAACAGCCAGCTGCACACCgtgaccaacTTCTTCCTGAGCAACCTGAGCCTGGTGGACCTGTGCTACAGCAGCGTGATCACCCCCAAGATGCTGATCAACTTCGTGAGCCAGAGAAACCTGATCAGCTACGTGGGCTGCATGAGCCAGCTGTACTTCTTCCTGGTGTTCGTGATCGCCGAGTGCTACATGCTGACCGTGATGGCCTACGACAGATACGTGGCCATCTGCCAGCCCCTGCTGTACAACATCATCATGAGCCCCGCCCTGTGCAGCCTGCTGGTGGCCTTCGTGTACGCCGTGGGCCTGATCGGCAGCGCCATCGAGACCGGCCTGATGCTGAAGCTGAACTACTGCGAGGACCTGATCAGCCACTACTTCTGCGACATCCTGCCCCTGATGAAGCTGAGCTGCAGCAGCACCTACGACGTGGAGATGGCCGTGTTCTTCCTGGCCGGCTTCGACATCATCGTGACCAGCCTGACCGTGCTGATCAGCTACGCCTTCATCCTGAGCAGCATCCTGAGAATCAGCAGCAACGAGGGCAGAAGCAAGGCCTTCAGCACCTGCAGCAGCCACTTCGCCGCCGTGGGCCTGTTCTACGGCAGCACCGCCTTCATGTACCTGAAGCCCAGCACCGCCAGCAGCCTGGCCCAGGAGAACGTGGCCAGCGTGTTCTACACCACCGTGATCCCCATGTTCAACCCCCTGATCTACAGCCTGAGAAACAAGGAGGTGAAGACCGCCCTGGACAAGACCCTGAGAAGAAAGGTGTTCTTAATTAA

**D1104: M71 M98E**

ATGACCGCCGAGAACCAGAGCACCGTGACCGAGTTCATCCTGGGCGGCCTGACCAACAGACCCGAGCTGCAGCTGCCCCTGTTCCTGCTGTTCCTGGGCATCTACGTGGTGACCATGGTGGGCAACCTGGGCATGATCACCCTGATCGGCCTGAACAGCCAGCTGCACACCCCCATGTACTTCTTCCTGAGCAACCTGAGCCTGGTGGACCTGTGCTACAGCAGCGTGATCACCCCCAAGATGCTGATCAACTTCGTGAGCCAGAGAAACCTGATCAGCTACGTGGGCTGCgaGAGCCAGCTGTACTTCTTCCTGGTGTTCGTGATCGCCGAGTGCTACATGCTGACCGTGATGGCCTACGACAGATACGTGGCCATCTGCCAGCCCCTGCTGTACAACATCATCATGAGCCCCGCCCTGTGCAGCCTGCTGGTGGCCTTCGTGTACGCCGTGGGCCTGATCGGCAGCGCCATCGAGACCGGCCTGATGCTGAAGCTGAACTACTGCGAGGACCTGATCAGCCACTACTTCTGCGACATCCTGCCCCTGATGAAGCTGAGCTGCAGCAGCACCTACGACGTGGAGATGGCCGTGTTCTTCCTGGCCGGCTTCGACATCATCGTGACCAGCCTGACCGTGCTGATCAGCTACGCCTTCATCCTGAGCAGCATCCTGAGAATCAGCAGCAACGAGGGCAGAAGCAAGGCCTTCAGCACCTGCAGCAGCCACTTCGCCGCCGTGGGCCTGTTCTACGGCAGCACCGCCTTCATGTACCTGAAGCCCAGCACCGCCAGCAGCCTGGCCCAGGAGAACGTGGCCAGCGTGTTCTACACCACCGTGATCCCCATGTTCAACCCCCTGATCTACAGCCTGAGAAACAAGGAGGTGAAGACCGCCCTGGACAAGACCCTGAGAAGAAAGGTGTTCTTAATTAA

**D1103: M71 C169A**

ATGACCGCCGAGAACCAGAGCACCGTGACCGAGTTCATCCTGGGCGGCCTGACCAACAGACCCGAGCTGCAGCTGCCCCTGTTCCTGCTGTTCCTGGGCATCTACGTGGTGACCATGGTGGGCAACCTGGGCATGATCACCCTGATCGGCCTGAACAGCCAGCTGCACACCCCCATGTACTTCTTCCTGAGCAACCTGAGCCTGGTGGACCTGTGCTACAGCAGCGTGATCACCCCCAAGATGCTGATCAACTTCGTGAGCCAGAGAAACCTGATCAGCTACGTGGGCTGCATGAGCCAGCTGTACTTCTTCCTGGTGTTCGTGATCGCCGAGTGCTACATGCTGACCGTGATGGCCTACGACAGATACGTGGCCATCTGCCAGCCCCTGCTGTACAACATCATCATGAGCCCCGCCCTGTGCAGCCTGCTGGTGGCCTTCGTGTACGCCGTGGGCCTGATCGGCAGCGCCATCGAGACCGGCCTGATGCTGAAGCTGAACTACgcCGAGGACCTGATCAGCCACTACTTCTGCGACATCCTGCCCCTGATGAAGCTGAGCTGCAGCAGCACCTACGACGTGGAGATGGCCGTGTTCTTCCTGGCCGGCTTCGACATCATCGTGACCAGCCTGACCGTGCTGATCAGCTACGCCTTCATCCTGAGCAGCATCCTGAGAATCAGCAGCAACGAGGGCAGAAGCAAGGCCTTCAGCACCTGCAGCAGCCACTTCGCCGCCGTGGGCCTGTTCTACGGCAGCACCGCCTTCATGTACCTGAAGCCCAGCACCGCCAGCAGCCTGGCCCAGGAGAACGTGGCCAGCGTGTTCTACACCACCGTGATCCCCATGTTCAACCCCCTGATCTACAGCCTGAGAAACAAGGAGGTGAAGACCGCCCTGGACAAGACCCTGAGAAGAAAGGTGTTCTTAATTAA

**D1102: M71 YF to TC**

ATGACCGCCGAGAACCAGAGCACCGTGACCGAGTTCATCCTGGGCGGCCTGACCAACAGACCCGAGCTGCAGCTGCCCCTGTTCCTGCTGTTCCTGGGCATCTACGTGGTGACCATGGTGGGCAACCTGGGCATGATCACCCTGATCGGCCTGAACAGCCAGCTGCACACCCCCATGTACTTCTTCCTGAGCAACCTGAGCCTGGTGGACCTGTGCTACAGCAGCGTGATCACCCCCAAGATGCTGATCAACTTCGTGAGCCAGAGAAACCTGATCAGCTACGTGGGCTGCATGAGCCAGCTGTACTTCTTCCTGGTGTTCGTGATCGCCGAGTGCTACATGCTGACCGTGATGGCCTACGACAGATACGTGGCCATCTGCCAGCCCCTGCTGTACAACATCATCATGAGCCCCGCCCTGTGCAGCCTGCTGGTGGCCTTCGTGTACGCCGTGGGCCTGATCGGCAGCGCCATCGAGACCGGCCTGATGCTGAAGCTGAACTACTGCGAGGACCTGATCAGCCACacCTgCTGCGACATCCTGCCCCTGATGAAGCTGAGCTGCAGCAGCACCTACGACGTGGAGATGGCCGTGTTCTTCCTGGCCGGCTTCGACATCATCGTGACCAGCCTGACCGTGCTGATCAGCTACGCCTTCATCCTGAGCAGCATCCTGAGAATCAGCAGCAACGAGGGCAGAAGCAAGGCCTTCAGCACCTGCAGCAGCCACTTCGCCGCCGTGGGCCTGTTCTACGGCAGCACCGCCTTCATGTACCTGAAGCCCAGCACCGCCAGCAGCCTGGCCCAGGAGAACGTGGCCAGCGTGTTCTACACCACCGTGATCCCCATGTTCAACCCCCTGATCTACAGCCTGAGAAACAAGGAGGTGAAGACCGCCCTGGACAAGACCCTGAGAAGAAAGGTGTTCTTAATTAA

**D1101: M71 C178A**

ATGACCGCCGAGAACCAGAGCACCGTGACCGAGTTCATCCTGGGCGGCCTGACCAACAGACCCGAGCTGCAGCTGCCCCTGTTCCTGCTGTTCCTGGGCATCTACGTGGTGACCATGGTGGGCAACCTGGGCATGATCACCCTGATCGGCCTGAACAGCCAGCTGCACACCCCCATGTACTTCTTCCTGAGCAACCTGAGCCTGGTGGACCTGTGCTACAGCAGCGTGATCACCCCCAAGATGCTGATCAACTTCGTGAGCCAGAGAAACCTGATCAGCTACGTGGGCTGCATGAGCCAGCTGTACTTCTTCCTGGTGTTCGTGATCGCCGAGTGCTACATGCTGACCGTGATGGCCTACGACAGATACGTGGCCATCTGCCAGCCCCTGCTGTACAACATCATCATGAGCCCCGCCCTGTGCAGCCTGCTGGTGGCCTTCGTGTACGCCGTGGGCCTGATCGGCAGCGCCATCGAGACCGGCCTGATGCTGAAGCTGAACTACTGCGAGGACCTGATCAGCCACTACTTCgcCGACATCCTGCCCCTGATGAAGCTGAGCTGCAGCAGCACCTACGACGTGGAGATGGCCGTGTTCTTCCTGGCCGGCTTCGACATCATCGTGACCAGCCTGACCGTGCTGATCAGCTACGCCTTCATCCTGAGCAGCATCCTGAGAATCAGCAGCAACGAGGGCAGAAGCAAGGCCTTCAGCACCTGCAGCAGCCACTTCGCCGCCGTGGGCCTGTTCTACGGCAGCACCGCCTTCATGTACCTGAAGCCCAGCACCGCCAGCAGCCTGGCCCAGGAGAACGTGGCCAGCGTGTTCTACACCACCGTGATCCCCATGTTCAACCCCCTGATCTACAGCCTGAGAAACAAGGAGGTGAAGACCGCCCTGGACAAGACCCTGAGAAGAAAGGTGTTCTTAATTAA

**D367: Y217A**

ATGACCGCCGAGAACCAGAGCACCGTGACCGAGTTCATCCTGGGCGGCCTGACCAACAGACCCGAGCTGCAGCTGCCCCTGTTCCTGCTGTTCCTGGGCATCTACGTGGTGACCATGGTGGGCAACCTGGGCATGATCACCCTGATCGGCCTGAACAGCCAGCTGCACACCCCCATGTACTTCTTCCTGAGCAACCTGAGCCTGGTGGACCTGTGCTACAGCAGCGTGATCACCCCCAAGATGCTGATCAACTTCGTGAGCCAGAGAAACCTGATCAGCTACGTGGGCTGCATGAGCCAGCTGTACTTCTTCCTGGTGTTCGTGATCGCCGAGTGCTACATGCTGACCGTGATGGCCTACGACAGATACGTGGCCATCTGCCAGCCCCTGCTGTACAACATCATCATGAGCCCCGCCCTGTGCAGCCTGCTGGTGGCCTTCGTGTACGCCGTGGGCCTGATCGGCAGCGCCATCGAGACCGGCCTGATGCTGAAGCTGAACTACTGCGAGGACCTGATCAGCCACTACTTCTGCGACATCCTGCCCCTGATGAAGCTGAGCTGCAGCAGCACCTACGACGTGGAGATGGCCGTGTTCTTCCTGGCCGGCTTCGACATCATCGTGACCAGCCTGACCGTGCTGATCAGCgcCGCCTTCATCCTGAGCAGCATCCTGAGAATCAGCAGCAACGAGGGCAGAAGCAAGGCCTTCAGCACCTGCAGCAGCCACTTCGCCGCCGTGGGCCTGTTCTACGGCAGCACCGCCTTCATGTACCTGAAGCCCAGCACCGCCAGCAGCCTGGCCCAGGAGAACGTGGCCAGCGTGTTCTACACCACCGTGATCCCCATGTTCAACCCCCTGATCTACAGCCTGAGAAACAAGGAGGTGAAGACCGCCCTGGACAAGACCCTGAGAAGAAAGGTGTTCTTAATTAA

**D385: M71 with 4 NQS copies**

ATGACCGCCGAGaacAGTagcaatAGCtctaacAGTagcaatAGCtctACCGTGACCGAGTTCATCCTGGGCGGCCTGACCAACAGACCCGAGCTGCAGCTGCCCCTGTTCCTGCTGTTCCTGGGCATCTACGTGGTGACCATGGTGGGCAACCTGGGCATGATCACCCTGATCGGCCTGAACAGCCAGCTGCACACCCCCATGTACTTCTTCCTGAGCAACCTGAGCCTGGTGGACCTGTGCTACAGCAGCGTGATCACCCCCAAGATGCTGATCAACTTCGTGAGCCAGAGAAACCTGATCAGCTACGTGGGCTGCATGAGCCAGCTGTACTTCTTCCTGGTGTTCGTGATCGCCGAGTGCTACATGCTGACCGTGATGGCCTACGACAGATACGTGGCCATCTGCCAGCCCCTGCTGTACAACATCATCATGAGCCCCGCCCTGTGCAGCCTGCTGGTGGCCTTCGTGTACGCCGTGGGCCTGATCGGCAGCGCCATCGAGACCGGCCTGATGCTGAAGCTGAACTACTGCGAGGACCTGATCAGCCACTACTTCTGCGACATCCTGCCCCTGATGAAGCTGAGCTGCAGCAGCACCTACGACGTGGAGATGGCCGTGTTCTTCCTGGCCGGCTTCGACATCATCGTGACCAGCCTGACCGTGCTGATCAGCTACGCCTTCATCCTGAGCAGCATCCTGAGAATCAGCAGCAACGAGGGCAGAAGCAAGGCCTTCAGCACCTGCAGCAGCCACTTCGCCGCCGTGGGCCTGTTCTACGGCAGCACCGCCTTCATGTACCTGAAGCCCAGCACCGCCAGCAGCCTGGCCCAGGAGAACGTGGCCAGCGTGTTCTACACCACCGTGATCCCCATGTTCAACCCCCTGATCTACAGCCTGAGAAACAAGGAGGTGAAGACCGCCCTGGACAAGACCCTGAGAAGAAAGGTGTTCTTAATTAA

**D1105: M71 with 4 NSS**

ATGACCGCCGAGaaccagagcaatcaatctaaccagagcaatcaatctACCGTGACCGAGTTCATCCTGGGCGGCCTGACCAACAGACCCGAGCTGCAGCTGCCCCTGTTCCTGCTGTTCCTGGGCATCTACGTGGTGACCATGGTGGGCAACCTGGGCATGATCACCCTGATCGGCCTGAACAGCCAGCTGCACACCCCCATGTACTTCTTCCTGAGCAACCTGAGCCTGGTGGACCTGTGCTACAGCAGCGTGATCACCCCCAAGATGCTGATCAACTTCGTGAGCCAGAGAAACCTGATCAGCTACGTGGGCTGCATGAGCCAGCTGTACTTCTTCCTGGTGTTCGTGATCGCCGAGTGCTACATGCTGACCGTGATGGCCTACGACAGATACGTGGCCATCTGCCAGCCCCTGCTGTACAACATCATCATGAGCCCCGCCCTGTGCAGCCTGCTGGTGGCCTTCGTGTACGCCGTGGGCCTGATCGGCAGCGCCATCGAGACCGGCCTGATGCTGAAGCTGAACTACTGCGAGGACCTGATCAGCCACTACTTCTGCGACATCCTGCCCCTGATGAAGCTGAGCTGCAGCAGCACCTACGACGTGGAGATGGCCGTGTTCTTCCTGGCCGGCTTCGACATCATCGTGACCAGCCTGACCGTGCTGATCAGCTACGCCTTCATCCTGAGCAGCATCCTGAGAATCAGCAGCAACGAGGGCAGAAGCAAGGCCTTCAGCACCTGCAGCAGCCACTTCGCCGCCGTGGGCCTGTTCTACGGCAGCACCGCCTTCATGTACCTGAAGCCCAGCACCGCCAGCAGCCTGGCCCAGGAGAACGTGGCCAGCGTGTTCTACACCACCGTGATCCCCATGTTCAACCCCCTGATCTACAGCCTGAGAAACAAGGAGGTGAAGACCGCCCTGGACAAGACCCTGAGAAGAAAGGTGTTCTTAATTAA

**D1109: M71 (no NXS) with NGT from Rhodopsin**

ATGaacggcaccgagACCGCCGAGCAGAGCACCGTGACCGAGTTCATCCTGGGCGGCCTGACCAACAGACCCGAGCTGCAGCTGCCCCTGTTCCTGCTGTTCCTGGGCATCTACGTGGTGACCATGGTGGGCAACCTGGGCATGATCACCCTGATCGGCCTGAACAGCCAGCTGCACACCCCCATGTACTTCTTCCTGAGCAACCTGAGCCTGGTGGACCTGTGCTACAGCAGCGTGATCACCCCCAAGATGCTGATCAACTTCGTGAGCCAGAGAAACCTGATCAGCTACGTGGGCTGCATGAGCCAGCTGTACTTCTTCCTGGTGTTCGTGATCGCCGAGTGCTACATGCTGACCGTGATGGCCTACGACAGATACGTGGCCATCTGCCAGCCCCTGCTGTACAACATCATCATGAGCCCCGCCCTGTGCAGCCTGCTGGTGGCCTTCGTGTACGCCGTGGGCCTGATCGGCAGCGCCATCGAGACCGGCCTGATGCTGAAGCTGAACTACTGCGAGGACCTGATCAGCCACTACTTCTGCGACATCCTGCCCCTGATGAAGCTGAGCTGCAGCAGCACCTACGACGTGGAGATGGCCGTGTTCTTCCTGGCCGGCTTCGACATCATCGTGACCAGCCTGACCGTGCTGATCAGCTACGCCTTCATCCTGAGCAGCATCCTGAGAATCAGCAGCAACGAGGGCAGAAGCAAGGCCTTCAGCACCTGCAGCAGCCACTTCGCCGCCGTGGGCCTGTTCTACGGCAGCACCGCCTTCATGTACCTGAAGCCCAGCACCGCCAGCAGCCTGGCCCAGGAGAACGTGGCCAGCGTGTTCTACACCACCGTGATCCCCATGTTCAACCCCCTGATCTACAGCCTGAGAAACAAGGAGGTGAAGACCGCCCTGGACAAGACCCTGAGAAGAAAGGTGTTCTTAATTAA

**D1110: M71 (no NXS) with NAT from Rhodopsin**

ATGaacgccaccggcACCGCCGAGCAGAGCACCGTGACCGAGTTCATCCTGGGCGGCCTGACCAACAGACCCGAGCTGCAGCTGCCCCTGTTCCTGCTGTTCCTGGGCATCTACGTGGTGACCATGGTGGGCAACCTGGGCATGATCACCCTGATCGGCCTGAACAGCCAGCTGCACACCCCCATGTACTTCTTCCTGAGCAACCTGAGCCTGGTGGACCTGTGCTACAGCAGCGTGATCACCCCCAAGATGCTGATCAACTTCGTGAGCCAGAGAAACCTGATCAGCTACGTGGGCTGCATGAGCCAGCTGTACTTCTTCCTGGTGTTCGTGATCGCCGAGTGCTACATGCTGACCGTGATGGCCTACGACAGATACGTGGCCATCTGCCAGCCCCTGCTGTACAACATCATCATGAGCCCCGCCCTGTGCAGCCTGCTGGTGGCCTTCGTGTACGCCGTGGGCCTGATCGGCAGCGCCATCGAGACCGGCCTGATGCTGAAGCTGAACTACTGCGAGGACCTGATCAGCCACTACTTCTGCGACATCCTGCCCCTGATGAAGCTGAGCTGCAGCAGCACCTACGACGTGGAGATGGCCGTGTTCTTCCTGGCCGGCTTCGACATCATCGTGACCAGCCTGACCGTGCTGATCAGCTACGCCTTCATCCTGAGCAGCATCCTGAGAATCAGCAGCAACGAGGGCAGAAGCAAGGCCTTCAGCACCTGCAGCAGCCACTTCGCCGCCGTGGGCCTGTTCTACGGCAGCACCGCCTTCATGTACCTGAAGCCCAGCACCGCCAGCAGCCTGGCCCAGGAGAACGTGGCCAGCGTGTTCTACACCACCGTGATCCCCATGTTCAACCCCCTGATCTACAGCCTGAGAAACAAGGAGGTGAAGACCGCCCTGGACAAGACCCTGAGAAGAAAGGTGTTCTTAATTAA

**D1106: Rhodopsin tagged M71**

atgaacggcaccgagggccccaacttctacgtgcccttcagcaacgccaccggcgtggtgagaATGACCGCCGAGAACCAGAGCACCGTGACCGAGTTCATCCTGGGCGGCCTGACCAACAGACCCGAGCTGCAGCTGCCCCTGTTCCTGCTGTTCCTGGGCATCTACGTGGTGACCATGGTGGGCAACCTGGGCATGATCACCCTGATCGGCCTGAACAGCCAGCTGCACACCCCCATGTACTTCTTCCTGAGCAACCTGAGCCTGGTGGACCTGTGCTACAGCAGCGTGATCACCCCCAAGATGCTGATCAACTTCGTGAGCCAGAGAAACCTGATCAGCTACGTGGGCTGCATGAGCCAGCTGTACTTCTTCCTGGTGTTCGTGATCGCCGAGTGCTACATGCTGACCGTGATGGCCTACGACAGATACGTGGCCATCTGCCAGCCCCTGCTGTACAACATCATCATGAGCCCCGCCCTGTGCAGCCTGCTGGTGGCCTTCGTGTACGCCGTGGGCCTGATCGGCAGCGCCATCGAGACCGGCCTGATGCTGAAGCTGAACTACTGCGAGGACCTGATCAGCCACTACTTCTGCGACATCCTGCCCCTGATGAAGCTGAGCTGCAGCAGCACCTACGACGTGGAGATGGCCGTGTTCTTCCTGGCCGGCTTCGACATCATCGTGACCAGCCTGACCGTGCTGATCAGCTACGCCTTCATCCTGAGCAGCATCCTGAGAATCAGCAGCAACGAGGGCAGAAGCAAGGCCTTCAGCACCTGCAGCAGCCACTTCGCCGCCGTGGGCCTGTTCTACGGCAGCACCGCCTTCATGTACCTGAAGCCCAGCACCGCCAGCAGCCTGGCCCAGGAGAACGTGGCCAGCGTGTTCTACACCACCGTGATCCCCATGTTCAACCCCCTGATCTACAGCCTGAGAAACAAGGAGGTGAAGACCGCCCTGGACAAGACCCTGAGAAGAAAGGTGTTCTTAATTAA

**D1108: M71 with K4 Nt**

atggcagcaggaaaccactgcACCGTGACCGAGTTCATCCTGGGCGGCCTGACCAACAGACCCGAGCTGCAGCTGCCCCTGTTCCTGCTGTTCCTGGGCATCTACGTGGTGACCATGGTGGGCAACCTGGGCATGATCACCCTGATCGGCCTGAACAGCCAGCTGCACACCCCCATGTACTTCTTCCTGAGCAACCTGAGCCTGGTGGACCTGTGCTACAGCAGCGTGATCACCCCCAAGATGCTGATCAACTTCGTGAGCCAGAGAAACCTGATCAGCTACGTGGGCTGCATGAGCCAGCTGTACTTCTTCCTGGTGTTCGTGATCGCCGAGTGCTACATGCTGACCGTGATGGCCTACGACAGATACGTGGCCATCTGCCAGCCCCTGCTGTACAACATCATCATGAGCCCCGCCCTGTGCAGCCTGCTGGTGGCCTTCGTGTACGCCGTGGGCCTGATCGGCAGCGCCATCGAGACCGGCCTGATGCTGAAGCTGAACTACTGCGAGGACCTGATCAGCCACTACTTCTGCGACATCCTGCCCCTGATGAAGCTGAGCTGCAGCAGCACCTACGACGTGGAGATGGCCGTGTTCTTCCTGGCCGGCTTCGACATCATCGTGACCAGCCTGACCGTGCTGATCAGCTACGCCTTCATCCTGAGCAGCATCCTGAGAATCAGCAGCAACGAGGGCAGAAGCAAGGCCTTCAGCACCTGCAGCAGCCACTTCGCCGCCGTGGGCCTGTTCTACGGCAGCACCGCCTTCATGTACCTGAAGCCCAGCACCGCCAGCAGCCTGGCCCAGGAGAACGTGGCCAGCGTGTTCTACACCACCGTGATCCCCATGTTCAACCCCCTGATCTACAGCCTGAGAAACAAGGAGGTGAAGACCGCCCTGGACAAGACCCTGAGAAGAAAGGTGTTCTTAATTAA

**D1107: Kirrel2**

atgctgagaatgagagtgcccgccctgctggtgctgctgttctgcttcagaggcagagccACCGCCGAGAACCAGAGCACCGTGACCGAGTTCATCCTGGGCGGCCTGACCAACAGACCCGAGCTGCAGCTGCCCCTGTTCCTGCTGTTCCTGGGCATCTACGTGGTGACCATGGTGGGCAACCTGGGCATGATCACCCTGATCGGCCTGAACAGCCAGCTGCACACCCCCATGTACTTCTTCCTGAGCAACCTGAGCCTGGTGGACCTGTGCTACAGCAGCGTGATCACCCCCAAGATGCTGATCAACTTCGTGAGCCAGAGAAACCTGATCAGCTACGTGGGCTGCATGAGCCAGCTGTACTTCTTCCTGGTGTTCGTGATCGCCGAGTGCTACATGCTGACCGTGATGGCCTACGACAGATACGTGGCCATCTGCCAGCCCCTGCTGTACAACATCATCATGAGCCCCGCCCTGTGCAGCCTGCTGGTGGCCTTCGTGTACGCCGTGGGCCTGATCGGCAGCGCCATCGAGACCGGCCTGATGCTGAAGCTGAACTACTGCGAGGACCTGATCAGCCACTACTTCTGCGACATCCTGCCCCTGATGAAGCTGAGCTGCAGCAGCACCTACGACGTGGAGATGGCCGTGTTCTTCCTGGCCGGCTTCGACATCATCGTGACCAGCCTGACCGTGCTGATCAGCTACGCCTTCATCCTGAGCAGCATCCTGAGAATCAGCAGCAACGAGGGCAGAAGCAAGGCCTTCAGCACCTGCAGCAGCCACTTCGCCGCCGTGGGCCTGTTCTACGGCAGCACCGCCTTCATGTACCTGAAGCCCAGCACCGCCAGCAGCCTGGCCCAGGAGAACGTGGCCAGCGTGTTCTACACCACCGTGATCCCCATGTTCAACCCCCTGATCTACAGCCTGAGAAACAAGGAGGTGAAGACCGCCCTGGACAAGACCCTGAGAAGAAAGGTGTTCTTAATTAA

**D1111: M71 with Calumenin tag** atggacctgagacagttcctgatgtgcctgagcctgtgcaccgccttcgccctgagcaagcccaccgagaagaaggacagagtgcaccacACCGCCGAGAACCAGAGCACCGTGACCGAGTTCATCCTGGGCGGCCTGACCAACAGACCCGAGCTGCAGCTGCCCCTGTTCCTGCTGTTCCTGGGCATCTACGTGGTGACCATGGTGGGCAACCTGGGCATGATCACCCTGATCGGCCTGAACAGCCAGCTGCACACCCCCATGTACTTCTTCCTGAGCAACCTGAGCCTGGTGGACCTGTGCTACAGCAGCGTGATCACCCCCAAGATGCTGATCAACTTCGTGAGCCAGAGAAACCTGATCAGCTACGTGGGCTGCATGAGCCAGCTGTACTTCTTCCTGGTGTTCGTGATCGCCGAGTGCTACATGCTGACCGTGATGGCCTACGACAGATACGTGGCCATCTGCCAGCCCCTGCTGTACAACATCATCATGAGCCCCGCCCTGTGCAGCCTGCTGGTGGCCTTCGTGTACGCCGTGGGCCTGATCGGCAGCGCCATCGAGACCGGCCTGATGCTGAAGCTGAACTACTGCGAGGACCTGATCAGCCACTACTTCTGCGACATCCTGCCCCTGATGAAGCTGAGCTGCAGCAGCACCTACGACGTGGAGATGGCCGTGTTCTTCCTGGCCGGCTTCGACATCATCGTGACCAGCCTGACCGTGCTGATCAGCTACGCCTTCATCCTGAGCAGCATCCTGAGAATCAGCAGCAACGAGGGCAGAAGCAAGGCCTTCAGCACCTGCAGCAGCCACTTCGCCGCCGTGGGCCTGTTCTACGGCAGCACCGCCTTCATGTACCTGAAGCCCAGCACCGCCAGCAGCCTGGCCCAGGAGAACGTGGCCAGCGTGTTCTACACCACCGTGATCCCCATGTTCAACCCCCTGATCTACAGCCTGAGAAACAAGGAGGTGAAGACCGCCCTGGACAAGACCCTGAGAAGAAAGGTGTTCTTAATTAA

**D1746: Endothelin Receptor B tag-M71**

atgcagcctcctcctagcctgtgcggcagggccctggtggccctggtgctggcctgcggcctgagcaggatctggggcACCGCCGAGAACCAGAGCACCGTGACCGAGTTCATCCTGGGCGGCCTGACCAACAGACCCGAGCTGCAGCTGCCCCTGTTCCTGCTGTTCCTGGGCATCTACGTGGTGACCATGGTGGGCAACCTGGGCATGATCACCCTGATCGGCCTGAACAGCCAGCTGCACACCCCCATGTACTTCTTCCTGAGCAACCTGAGCCTGGTGGACCTGTGCTACAGCAGCGTGATCACCCCCAAGATGCTGATCAACTTCGTGAGCCAGAGAAACCTGATCAGCTACGTGGGCTGCATGAGCCAGCTGTACTTCTTCCTGGTGTTCGTGATCGCCGAGTGCTACATGCTGACCGTGATGGCCTACGACAGATACGTGGCCATCTGCCAGCCCCTGCTGTACAACATCATCATGAGCCCCGCCCTGTGCAGCCTGCTGGTGGCCTTCGTGTACGCCGTGGGCCTGATCGGCAGCGCCATCGAGACCGGCCTGATGCTGAAGCTGAACTACTGCGAGGACCTGATCAGCCACTACTTCTGCGACATCCTGCCCCTGATGAAGCTGAGCTGCAGCAGCACCTACGACGTGGAGATGGCCGTGTTCTTCCTGGCCGGCTTCGACATCATCGTGACCAGCCTGACCGTGCTGATCAGCTACGCCTTCATCCTGAGCAGCATCCTGAGAATCAGCAGCAACGAGGGCAGAAGCAAGGCCTTCAGCACCTGCAGCAGCCACTTCGCCGCCGTGGGCCTGTTCTACGGCAGCACCGCCTTCATGTACCTGAAGCCCAGCACCGCCAGCAGCCTGGCCCAGGAGAACGTGGCCAGCGTGTTCTACACCACCGTGATCCCCATGTTCAACCCCCTGATCTACAGCCTGAGAAACAAGGAGGTGAAGACCGCCCTGGACAAGACCCTGAGAAGAAAGGTGTTCTTAATTAA

**D1128: 5HT3 tag-M71**

atggtgctgtgggtgcagcaggccctgctggccctgctgctgcccaccctgctggcccagggcgaggccagaACCGCCGAGAACCAGAGCACCGTGACCGAGTTCATCCTGGGCGGCCTGACCAACAGACCCGAGCTGCAGCTGCCCCTGTTCCTGCTGTTCCTGGGCATCTACGTGGTGACCATGGTGGGCAACCTGGGCATGATCACCCTGATCGGCCTGAACAGCCAGCTGCACACCCCCATGTACTTCTTCCTGAGCAACCTGAGCCTGGTGGACCTGTGCTACAGCAGCGTGATCACCCCCAAGATGCTGATCAACTTCGTGAGCCAGAGAAACCTGATCAGCTACGTGGGCTGCATGAGCCAGCTGTACTTCTTCCTGGTGTTCGTGATCGCCGAGTGCTACATGCTGACCGTGATGGCCTACGACAGATACGTGGCCATCTGCCAGCCCCTGCTGTACAACATCATCATGAGCCCCGCCCTGTGCAGCCTGCTGGTGGCCTTCGTGTACGCCGTGGGCCTGATCGGCAGCGCCATCGAGACCGGCCTGATGCTGAAGCTGAACTACTGCGAGGACCTGATCAGCCACTACTTCTGCGACATCCTGCCCCTGATGAAGCTGAGCTGCAGCAGCACCTACGACGTGGAGATGGCCGTGTTCTTCCTGGCCGGCTTCGACATCATCGTGACCAGCCTGACCGTGCTGATCAGCTACGCCTTCATCCTGAGCAGCATCCTGAGAATCAGCAGCAACGAGGGCAGAAGCAAGGCCTTCAGCACCTGCAGCAGCCACTTCGCCGCCGTGGGCCTGTTCTACGGCAGCACCGCCTTCATGTACCTGAAGCCCAGCACCGCCAGCAGCCTGGCCCAGGAGAACGTGGCCAGCGTGTTCTACACCACCGTGATCCCCATGTTCAACCCCCTGATCTACAGCCTGAGAAACAAGGAGGTGAAGACCGCCCTGGACAAGACCCTGAGAAGAAAGGTGTTCTTAATTAA

**D379: mB2AR Nt on M71**

atgggcccccacggcaacgacagcgacttcctgctggcccccaacggcagcagagcccccgaccacgacgtgacccaggagagagacgaggcctgggtggtgggcCTGTTCCTGCTGTTCCTGGGCATCTACGTGGTGACCATGGTGGGCAACCTGGGCATGATCACCCTGATCGGCCTGAACAGCCAGCTGCACACCCCCATGTACTTCTTCCTGAGCAACCTGAGCCTGGTGGACCTGTGCTACAGCAGCGTGATCACCCCCAAGATGCTGATCAACTTCGTGAGCCAGAGAAACCTGATCAGCTACGTGGGCTGCATGAGCCAGCTGTACTTCTTCCTGGTGTTCGTGATCGCCGAGTGCTACATGCTGACCGTGATGGCCTACGACAGATACGTGGCCATCTGCCAGCCCCTGCTGTACAACATCATCATGAGCCCCGCCCTGTGCAGCCTGCTGGTGGCCTTCGTGTACGCCGTGGGCCTGATCGGCAGCGCCATCGAGACCGGCCTGATGCTGAAGCTGAACTACTGCGAGGACCTGATCAGCCACTACTTCTGCGACATCCTGCCCCTGATGAAGCTGAGCTGCAGCAGCACCTACGACGTGGAGATGGCCGTGTTCTTCCTGGCCGGCTTCGACATCATCGTGACCAGCCTGACCGTGCTGATCAGCTACGCCTTCATCCTGAGCAGCATCCTGAGAATCAGCAGCAACGAGGGCAGAAGCAAGGCCTTCAGCACCTGCAGCAGCCACTTCGCCGCCGTGGGCCTGTTCTACGGCAGCACCGCCTTCATGTACCTGAAGCCCAGCACCGCCAGCAGCCTGGCCCAGGAGAACGTGGCCAGCGTGTTCTACACCACCGTGATCCCCATGTTCAACCCCCTGATCTACAGCCTGAGAAACAAGGAGGTGAAGACCGCCCTGGACAAGACCCTGAGAAGAAAGGTGTTCTTAATTAA

**D1181-2: M71 with mB2AR Ct**

ATGACCGCCGAGAACCAGAGCACCGTGACCGAGTTCATCCTGGGCGGCCTGACCAACAGACCCGAGCTGCAGCTGCCCCTGTTCCTGCTGTTCCTGGGCATCTACGTGGTGACCATGGTGGGCAACCTGGGCATGATCACCCTGATCGGCCTGAACAGCCAGCTGCACACCCCCATGTACTTCTTCCTGAGCAACCTGAGCCTGGTGGACCTGTGCTACAGCAGCGTGATCACCCCCAAGATGCTGATCAACTTCGTGAGCCAGAGAAACCTGATCAGCTACGTGGGCTGCATGAGCCAGCTGTACTTCTTCCTGGTGTTCGTGATCGCCGAGTGCTACATGCTGACCGTGATGGCCTACGACAGATACGTGGCCATCTGCCAGCCCCTGCTGTACAACATCATCATGAGCCCCGCCCTGTGCAGCCTGCTGGTGGCCTTCGTGTACGCCGTGGGCCTGATCGGCAGCGCCATCGAGACCGGCCTGATGCTGAAGCTGAACTACTGCGAGGACCTGATCAGCCACTACTTCTGCGACATCCTGCCCCTGATGAAGCTGAGCTGCAGCAGCACCTACGACGTGGAGATGGCCGTGTTCTTCCTGGCCGGCTTCGACATCATCGTGACCAGCCTGACCGTGCTGATCAGCTACGCCTTCATCCTGAGCAGCATCCTGAGAATCAGCAGCAACGAGGGCAGAAGCAAGGCCTTCAGCACCTGCAGCAGCCACTTCGCCGCCGTGGGCCTGTTCTACGGCAGCACCGCCTTCATGTACCTGAAGCCCAGCACCGCCAGCAGCCTGGCCCAGGAGAACGTGGCCAGCGTGTTCTACACCACCGTGATCCCCATGTTCAACCCCCTGATCTACtgtcggagtccagatttcaggattgcctttcaagagcttctgtgccttcgcaggtcttcttcgaaaacctatTTAATTAA

**D1754: mB2AR Nt M71 mB2AR Ct**

atgggcccccacggcaacgacagcgacttcctgctggcccccaacggcagcagagcccccgaccacgacgtgacccaggagagagacgaggcctgggtggtgggcCTGTTCCTGCTGTTCCTGGGCATCTACGTGGTGACCATGGTGGGCAACCTGGGCATGATCACCCTGATCGGCCTGAACAGCCAGCTGCACACCCCCATGTACTTCTTCCTGAGCAACCTGAGCCTGGTGGACCTGTGCTACAGCAGCGTGATCACCCCCAAGATGCTGATCAACTTCGTGAGCCAGAGAAACCTGATCAGCTACGTGGGCTGCATGAGCCAGCTGTACTTCTTCCTGGTGTTCGTGATCGCCGAGTGCTACATGCTGACCGTGATGGCCTACGACAGATACGTGGCCATCTGCCAGCCCCTGCTGTACAACATCATCATGAGCCCCGCCCTGTGCAGCCTGCTGGTGGCCTTCGTGTACGCCGTGGGCCTGATCGGCAGCGCCATCGAGACCGGCCTGATGCTGAAGCTGAACTACTGCGAGGACCTGATCAGCCACTACTTCTGCGACATCCTGCCCCTGATGAAGCTGAGCTGCAGCAGCACCTACGACGTGGAGATGGCCGTGTTCTTCCTGGCCGGCTTCGACATCATCGTGACCAGCCTGACCGTGCTGATCAGCTACGCCTTCATCCTGAGCAGCATCCTGAGAATCAGCAGCAACGAGGGCAGAAGCAAGGCCTTCAGCACCTGCAGCAGCCACTTCGCCGCCGTGGGCCTGTTCTACGGCAGCACCGCCTTCATGTACCTGAAGCCCAGCACCGCCAGCAGCCTGGCCCAGGAGAACGTGGCCAGCGTGTTCTACACCACCGTGATCCCCATGTTCAACCCCCTGATCTACtgtcggagtccagatttcaggattgcctttcaagagcttctgtgccttcgcaggtcttcttcgaaaacctatTTAATTAA

**D1753: all conserved mutations**

ATGACCGCCGAGAACCAGAGCACCGTGACCGAGgaccacgacgtGacCCTGACCAACAGACCCGAGCTGCAGCTGCCCCTGTTCCTGCTGTTCCTGGGCATCgcCGTGGTGACCATGGTGGGCAACCTGGGCATGATCACCCTGATCGGCCTGAACAGCCAGCTGCACACCgtgaccaACTTCTTCCTGAGCAACCTGAGCCTGGTGGACCTGTGCTACAGCAGCGTGATCACCCCCAAGATGCTGATCAACTTCGTGAGCCAGAGAAACCTGATCAGCTACGTGGGCTGCgaGAGCCAGCTGTACTTCTTCCTGGTGTTCGTGATCGCCGAGTGCTACATGCTGACCGTGATGGCCTACGACAGATACGTGGCCATCTGCCAGCCCCTGCTGTACAACATCATCATGAGCCCCGCCCTGTGCAGCCTGCTGGTGGCCTTCGTGTACGCCGTGGGCCTGATCGGCAGCGCCATCGAGACCGGCCTGATGCTGAAGCTGAACTACgcCGAGGACCTGATCAGCCACacCTgCgcCGACATCCTGCCCCTGATGAAGCTGAGCTGCAGCAGCACCTACGACGTGGAGATGGCCGTGTTCTTCCTGGCCGGCTTCGACATCATCGTGACCAGCCTGACCGTGCTGATCAGCgcCGCCTTCATCCTGAGCAGCATCCTGAGAATCAGCAGCAACGAGGGCAGAAGCAAGGCCTTCAGCACCTGCAGCAGCCACTTCGCCGCCGTGGGCCTGTTCTACGGCAGCACCGCCTTCATGTACCTGAAGCCCAGCACCGCCAGCAGCCTGGCCCAGGAGAACGTGGCCAGCGTGTTCTACACCACCGTGATCCCCATGTTCAACCCCCTGATCTACAGCCTGAGAAACAAGGAGGTGAAGACCGCCCTGGACAAGACCCTGAGAAGAAAGGTGTTCTTAATTAA

**D1743: LUCY-M71**

atgagaccccagatcctgctgctcctggccctgctgaccctaggcctggctACCGCCGAGAACCAGAGCACCGTGACCGAGTTCATCCTGGGCGGCCTGACCAACAGACCCGAGCTGCAGCTGCCCCTGTTCCTGCTGTTCCTGGGCATCTACGTGGTGACCATGGTGGGCAACCTGGGCATGATCACCCTGATCGGCCTGAACAGCCAGCTGCACACCCCCATGTACTTCTTCCTGAGCAACCTGAGCCTGGTGGACCTGTGCTACAGCAGCGTGATCACCCCCAAGATGCTGATCAACTTCGTGAGCCAGAGAAACCTGATCAGCTACGTGGGCTGCATGAGCCAGCTGTACTTCTTCCTGGTGTTCGTGATCGCCGAGTGCTACATGCTGACCGTGATGGCCTACGACAGATACGTGGCCATCTGCCAGCCCCTGCTGTACAACATCATCATGAGCCCCGCCCTGTGCAGCCTGCTGGTGGCCTTCGTGTACGCCGTGGGCCTGATCGGCAGCGCCATCGAGACCGGCCTGATGCTGAAGCTGAACTACTGCGAGGACCTGATCAGCCACTACTTCTGCGACATCCTGCCCCTGATGAAGCTGAGCTGCAGCAGCACCTACGACGTGGAGATGGCCGTGTTCTTCCTGGCCGGCTTCGACATCATCGTGACCAGCCTGACCGTGCTGATCAGCTACGCCTTCATCCTGAGCAGCATCCTGAGAATCAGCAGCAACGAGGGCAGAAGCAAGGCCTTCAGCACCTGCAGCAGCCACTTCGCCGCCGTGGGCCTGTTCTACGGCAGCACCGCCTTCATGTACCTGAAGCCCAGCACCGCCAGCAGCCTGGCCCAGGAGAACGTGGCCAGCGTGTTCTACACCACCGTGATCCCCATGTTCAACCCCCTGATCTACAGCCTGAGAAACAAGGAGGTGAAGACCGCCCTGGACAAGACCCTGAGAAGAAAGGTGTTCTTAATTAA

**D1744: LUCY-FLAG-M71**

atgagaccccagatcctgctgctcctggccctgctgaccctaggcctggctgattacaaggatgacgatgacaagACCGCCGAGAACCAGAGCACCGTGACCGAGTTCATCCTGGGCGGCCTGACCAACAGACCCGAGCTGCAGCTGCCCCTGTTCCTGCTGTTCCTGGGCATCTACGTGGTGACCATGGTGGGCAACCTGGGCATGATCACCCTGATCGGCCTGAACAGCCAGCTGCACACCCCCATGTACTTCTTCCTGAGCAACCTGAGCCTGGTGGACCTGTGCTACAGCAGCGTGATCACCCCCAAGATGCTGATCAACTTCGTGAGCCAGAGAAACCTGATCAGCTACGTGGGCTGCATGAGCCAGCTGTACTTCTTCCTGGTGTTCGTGATCGCCGAGTGCTACATGCTGACCGTGATGGCCTACGACAGATACGTGGCCATCTGCCAGCCCCTGCTGTACAACATCATCATGAGCCCCGCCCTGTGCAGCCTGCTGGTGGCCTTCGTGTACGCCGTGGGCCTGATCGGCAGCGCCATCGAGACCGGCCTGATGCTGAAGCTGAACTACTGCGAGGACCTGATCAGCCACTACTTCTGCGACATCCTGCCCCTGATGAAGCTGAGCTGCAGCAGCACCTACGACGTGGAGATGGCCGTGTTCTTCCTGGCCGGCTTCGACATCATCGTGACCAGCCTGACCGTGCTGATCAGCTACGCCTTCATCCTGAGCAGCATCCTGAGAATCAGCAGCAACGAGGGCAGAAGCAAGGCCTTCAGCACCTGCAGCAGCCACTTCGCCGCCGTGGGCCTGTTCTACGGCAGCACCGCCTTCATGTACCTGAAGCCCAGCACCGCCAGCAGCCTGGCCCAGGAGAACGTGGCCAGCGTGTTCTACACCACCGTGATCCCCATGTTCAACCCCCTGATCTACAGCCTGAGAAACAAGGAGGTGAAGACCGCCCTGGACAAGACCCTGAGAAGAAAGGTGTTCTTAATTAA

**D1735: Y289A in NPXXY M71**

ATGACCGCCGAGAACCAGAGCACCGTGACCGAGTTCATCCTGGGCGGCCTGACCAACAGACCCGAGCTGCAGCTGCCCCTGTTCCTGCTGTTCCTGGGCATCTACGTGGTGACCATGGTGGGCAACCTGGGCATGATCACCCTGATCGGCCTGAACAGCCAGCTGCACACCCCCATGTACTTCTTCCTGAGCAACCTGAGCCTGGTGGACCTGTGCTACAGCAGCGTGATCACCCCCAAGATGCTGATCAACTTCGTGAGCCAGAGAAACCTGATCAGCTACGTGGGCTGCATGAGCCAGCTGTACTTCTTCCTGGTGTTCGTGATCGCCGAGTGCTACATGCTGACCGTGATGGCCTACGACAGATACGTGGCCATCTGCCAGCCCCTGCTGTACAACATCATCATGAGCCCCGCCCTGTGCAGCCTGCTGGTGGCCTTCGTGTACGCCGTGGGCCTGATCGGCAGCGCCATCGAGACCGGCCTGATGCTGAAGCTGAACTACTGCGAGGACCTGATCAGCCACTACTTCTGCGACATCCTGCCCCTGATGAAGCTGAGCTGCAGCAGCACCTACGACGTGGAGATGGCCGTGTTCTTCCTGGCCGGCTTCGACATCATCGTGACCAGCCTGACCGTGCTGATCAGCTACGCCTTCATCCTGAGCAGCATCCTGAGAATCAGCAGCAACGAGGGCAGAAGCAAGGCCTTCAGCACCTGCAGCAGCCACTTCGCCGCCGTGGGCCTGTTCTACGGCAGCACCGCCTTCATGTACCTGAAGCCCAGCACCGCCAGCAGCCTGGCCCAGGAGAACGTGGCCAGCGTGTTCTACACCACCGTGATCCCCATGTTCAACCCCCTGATCgcCAGCCTGAGAAACAAGGAGGTGAAGACCGCCCTGGACAAGACCCTGAGAAGAAAGGTGTTCTGA

**D1736: Y289A in NPXXY no Ct**

ATGACCGCCGAGAACCAGAGCACCGTGACCGAGTTCATCCTGGGCGGCCTGACCAACAGACCCGAGCTGCAGCTGCCCCTGTTCCTGCTGTTCCTGGGCATCTACGTGGTGACCATGGTGGGCAACCTGGGCATGATCACCCTGATCGGCCTGAACAGCCAGCTGCACACCCCCATGTACTTCTTCCTGAGCAACCTGAGCCTGGTGGACCTGTGCTACAGCAGCGTGATCACCCCCAAGATGCTGATCAACTTCGTGAGCCAGAGAAACCTGATCAGCTACGTGGGCTGCATGAGCCAGCTGTACTTCTTCCTGGTGTTCGTGATCGCCGAGTGCTACATGCTGACCGTGATGGCCTACGACAGATACGTGGCCATCTGCCAGCCCCTGCTGTACAACATCATCATGAGCCCCGCCCTGTGCAGCCTGCTGGTGGCCTTCGTGTACGCCGTGGGCCTGATCGGCAGCGCCATCGAGACCGGCCTGATGCTGAAGCTGAACTACTGCGAGGACCTGATCAGCCACTACTTCTGCGACATCCTGCCCCTGATGAAGCTGAGCTGCAGCAGCACCTACGACGTGGAGATGGCCGTGTTCTTCCTGGCCGGCTTCGACATCATCGTGACCAGCCTGACCGTGCTGATCAGCTACGCCTTCATCCTGAGCAGCATCCTGAGAATCAGCAGCAACGAGGGCAGAAGCAAGGCCTTCAGCACCTGCAGCAGCCACTTCGCCGCCGTGGGCCTGTTCTACGGCAGCACCGCCTTCATGTACCTGAAGCCCAGCACCGCCAGCAGCCTGGCCCAGGAGAACGTGGCCAGCGTGTTCTACACCACCGTGATCCCCATGTTCAACCCCCTGATCgcCAGCCTGttaattaa

**D1737: mB2AR-Nt Y289A in NPXXY M71**

atgggcccccacggcaacgacagcgacttcctgctggcccccaacggcagcagagcccccgaccacgacgtgacccaggagagagacgaggcctgggtggtgggcCTGTTCCTGCTGTTCCTGGGCATCTACGTGGTGACCATGGTGGGCAACCTGGGCATGATCACCCTGATCGGCCTGAACAGCCAGCTGCACACCCCCATGTACTTCTTCCTGAGCAACCTGAGCCTGGTGGACCTGTGCTACAGCAGCGTGATCACCCCCAAGATGCTGATCAACTTCGTGAGCCAGAGAAACCTGATCAGCTACGTGGGCTGCATGAGCCAGCTGTACTTCTTCCTGGTGTTCGTGATCGCCGAGTGCTACATGCTGACCGTGATGGCCTACGACAGATACGTGGCCATCTGCCAGCCCCTGCTGTACAACATCATCATGAGCCCCGCCCTGTGCAGCCTGCTGGTGGCCTTCGTGTACGCCGTGGGCCTGATCGGCAGCGCCATCGAGACCGGCCTGATGCTGAAGCTGAACTACTGCGAGGACCTGATCAGCCACTACTTCTGCGACATCCTGCCCCTGATGAAGCTGAGCTGCAGCAGCACCTACGACGTGGAGATGGCCGTGTTCTTCCTGGCCGGCTTCGACATCATCGTGACCAGCCTGACCGTGCTGATCAGCTACGCCTTCATCCTGAGCAGCATCCTGAGAATCAGCAGCAACGAGGGCAGAAGCAAGGCCTTCAGCACCTGCAGCAGCCACTTCGCCGCCGTGGGCCTGTTCTACGGCAGCACCGCCTTCATGTACCTGAAGCCCAGCACCGCCAGCAGCCTGGCCCAGGAGAACGTGGCCAGCGTGTTCTACACCACCGTGATCCCCATGTTCAACCCCCTGATCgcCAGCCTGAGAAACAAGGAGGTGAAGACCGCCCTGGACAAGACCCTGAGAAGAAAGGTGTTC

**D1738: mB2AR-Nt Y289A in NPXXY M71 and no Ct**

atgggcccccacggcaacgacagcgacttcctgctggcccccaacggcagcagagcccccgaccacgacgtgacccaggagagagacgaggcctgggtggtgggcCTGTTCCTGCTGTTCCTGGGCATCTACGTGGTGACCATGGTGGGCAACCTGGGCATGATCACCCTGATCGGCCTGAACAGCCAGCTGCACACCCCCATGTACTTCTTCCTGAGCAACCTGAGCCTGGTGGACCTGTGCTACAGCAGCGTGATCACCCCCAAGATGCTGATCAACTTCGTGAGCCAGAGAAACCTGATCAGCTACGTGGGCTGCATGAGCCAGCTGTACTTCTTCCTGGTGTTCGTGATCGCCGAGTGCTACATGCTGACCGTGATGGCCTACGACAGATACGTGGCCATCTGCCAGCCCCTGCTGTACAACATCATCATGAGCCCCGCCCTGTGCAGCCTGCTGGTGGCCTTCGTGTACGCCGTGGGCCTGATCGGCAGCGCCATCGAGACCGGCCTGATGCTGAAGCTGAACTACTGCGAGGACCTGATCAGCCACTACTTCTGCGACATCCTGCCCCTGATGAAGCTGAGCTGCAGCAGCACCTACGACGTGGAGATGGCCGTGTTCTTCCTGGCCGGCTTCGACATCATCGTGACCAGCCTGACCGTGCTGATCAGCTACGCCTTCATCCTGAGCAGCATCCTGAGAATCAGCAGCAACGAGGGCAGAAGCAAGGCCTTCAGCACCTGCAGCAGCCACTTCGCCGCCGTGGGCCTGTTCTACGGCAGCACCGCCTTCATGTACCTGAAGCCCAGCACCGCCAGCAGCCTGGCCCAGGAGAACGTGGCCAGCGTGTTCTACACCACCGTGATCCCCATGTTCAACCCCCTGATCgcCAGCCTGTTAATTAA

**D1771: mB2AR-Nt Y289A and mB2AR Ct**

atgggcccccacggcaacgacagcgacttcctgctggcccccaacggcagcagagcccccgaccacgacgtgacccaggagagagacgaggcctgggtggtgggcCTGTTCCTGCTGTTCCTGGGCATCTACGTGGTGACCATGGTGGGCAACCTGGGCATGATCACCCTGATCGGCCTGAACAGCCAGCTGCACACCCCCATGTACTTCTTCCTGAGCAACCTGAGCCTGGTGGACCTGTGCTACAGCAGCGTGATCACCCCCAAGATGCTGATCAACTTCGTGAGCCAGAGAAACCTGATCAGCTACGTGGGCTGCATGAGCCAGCTGTACTTCTTCCTGGTGTTCGTGATCGCCGAGTGCTACATGCTGACCGTGATGGCCTACGACAGATACGTGGCCATCTGCCAGCCCCTGCTGTACAACATCATCATGAGCCCCGCCCTGTGCAGCCTGCTGGTGGCCTTCGTGTACGCCGTGGGCCTGATCGGCAGCGCCATCGAGACCGGCCTGATGCTGAAGCTGAACTACTGCGAGGACCTGATCAGCCACTACTTCTGCGACATCCTGCCCCTGATGAAGCTGAGCTGCAGCAGCACCTACGACGTGGAGATGGCCGTGTTCTTCCTGGCCGGCTTCGACATCATCGTGACCAGCCTGACCGTGCTGATCAGCTACGCCTTCATCCTGAGCAGCATCCTGAGAATCAGCAGCAACGAGGGCAGAAGCAAGGCCTTCAGCACCTGCAGCAGCCACTTCGCCGCCGTGGGCCTGTTCTACGGCAGCACCGCCTTCATGTACCTGAAGCCCAGCACCGCCAGCAGCCTGGCCCAGGAGAACGTGGCCAGCGTGTTCTACACCACCGTGATCCCCATGTTCAACCCCCTGATCgcCTGTCGGAGTCCAGATTTCAGGATTGCCTTTCAAGAGCTTCTGTGCCTTCGCAGGTCTTCTTCGAAAACCTAT

**OR1A1**

ATGAGGGAAAATAACCAGTCCTCTACACTGGAATTCATCCTCCTGGGAGTTACTGGTCAGCAGGAACAGGAAGATTTCTTCTACATCCTCTTCTTGTTCATTTACCCCATCACATTGATTGGAAACCTGCTCATCGTCCTAGCCATTTGCTCTGATGTTCGCCTTCACAACCCCATGTATTTTCTCCTTGCCAACCTCTCCTTGGTTGACATCTTCTTCTCATCGGTAACCATCCCTAAGATGCTGGCCAACCATCTCTTGGGCAGCAAATCCATCTCTTTTGGGGGATGCCTAACGCAGATGTATTTCATGATAGCCTTGGGTAACACAGACAGCTATATTTTGGCTGCAATGGCATATGATCGAGCTGTGGCCATCAGCCGCCCACTTCACTACACAACAATTATGAGTCCACGGTCTTGTATCTGGCTTATTGCTGGGTCTTGGGTGATTGGAAATGCCAATGCCCTCCCCCACACTCTGCTCACAGCTAGTCTGTCCTTCTGTGGCAACCAGGAAGTGGCCAACTTCTACTGTGACATTACCCCCTTGCTGAAGTTATCCTGTTCTGACATCCACTTTCATGTGAAGATGATGTACCTAGGGGTTGGCATTTTCTCTGTGCCATTACTATGCATCATTGTCTCCTATATTCGAGTCTTCTCCACAGTCTTCCAGGTTCCTTCCACCAAGGGCGTGCTCAAGGCCTTCTCCACCTGTGGTTCCCACCTCACGGTTGTCTCTTTGTATTATGGTACAGTCATGGGCACGTATTTCCGCCCTTTGACCAATTATAGCCTAAAAGACGCAGTGATCACTGTAATGTACACGGCAGTGACCCCAATGTTAAATCCTTTCATCTACAGTCTGAGAAATCGGGACATGAAGGCTGCCCTGCGGAAACTCTTCAACAAGAGAATCTCCTCGTAA

**Plasmids names and numbers:**

**D357: mB2AR**

**D1569-1: RTP1S**

**D358: M71**

**D371: M71 F12D I13H L14D G15V G16T**

**D372: M71 Y35A**

**D374: M71 PMY to VTN**

**D1104: M71 M98E**

**D1103: M71 C169A**

**D1102: M71 YF to TC**

**D1101: M71 C178A**

**D367: Y217A**

**D385: M71 with 4 NQS copies**

**D1105: M71 with 4 NSS**

**D1109: M71 (no NXS) with NGT from Rhodopsin**

**D1110: M71 (no NXS) with NAT from Rhodopsin**

**D1106: Rhodopsin tagged M71**

**D1108: M71 with K4 Nt**

**D1107: Kirrel2**

**D1111: M71 with Calumenin tag**

**D1746: Endothelin Receptor B tag-M71**

**D1128: 5HT3 tag-M71**

**D379: mB2AR Nt on M71**

**D1181-2: M71 with mB2AR Ct**

**D1754: mB2AR Nt M71 mB2AR Ct**

**D1753: all conserved mutations**

**D1743: LUCY-M71**

**D1744: LUCY-FLAG-M71**

**D1735: Y289A in NPXXY M71**

**D1736: Y289A in NPXXY no Ct**

**D1737: mB2AR-Nt Y289A in NPXXY M71**

**D1738: mB2AR-Nt Y289A in NPXXY M71 and no Ct**

**D1771: mB2AR-Nt Y289A and mB2AR Ct**

**D1796-4: OR1A1**
